# Supplementary material for: Synthesis and Biological Evaluation of Novel Amino and Amido Substituted Pentacyclic Benzimidazole Derivatives as Antiproliferative Agents
Source: Int J Mol Sci. 2024 Feb 14;25(4):2288. doi: 10.3390/ijms25042288 (PMC10889688; doi:10.3390/ijms25042288)
Supplement: Supplementary file 1 [file ijms-25-02288-s001.zip › ijms-2842409-supplementary.pdf]

Supporting information

Synthesis and biological evaluation of novel amino and amido substituted pentacyclic benzimidazole derivatives

Nataša Perin[1], Marjana Gulin[1], Marija Kos[1], Leentje Persoons[2], Dirk Daelmans[2], Ivana Fabijanić[3], Marijana Radić Stojković[3,*] and Marijana Hranjec [1,*]

[1] Department of Organic Chemistry, Faculty of Chemical Engineering and Technology, University of Zagreb, Marulićev trg 20, HR-10000 Zagreb, Croatia; [2] KU Leuven, Department of Microbiology and Immunology, Laboratory of Virology and Chemotherapy, Rega Institute, Leuven, Belgium; [3] Ruđer Bošković Institute, Division of Organic Chemistry and Biochemistry, Bijenička cesta 54, HR-10000 Zagreb, Croatia;

Contents

1. Spectroscopic characterization of pentacyclic benzimidazoles in aqueous solutions.
2. Interactions of pentacyclic benzimidazoles with ds-polynucleotides in neutral medium (pH=7.0)
2.1. Fluorimetric titrations
2.2. Thermal melting experiments
2.3. Circular dichroism (CD) titrations
3. NMR spectra of synthesized compounds (S18-S59)

1. Spectroscopic characterization of pentacyclic benzimidazoles in aqueous solutions

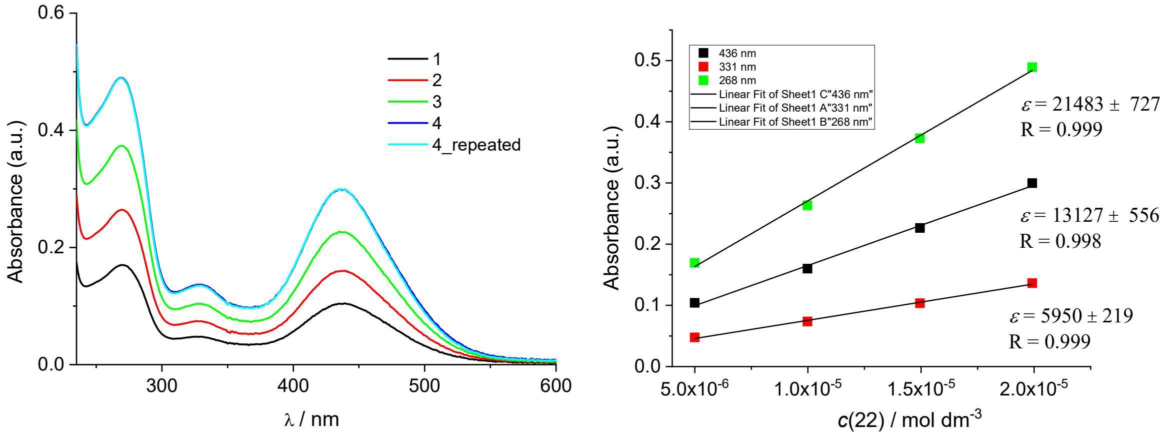

Figure S1. UV/Vis spectra changes of **22** at different concentrations (concentration range from 5.0×10^{-6} to 2.0×10^{-5} mol dm^{-3}) at pH=7.0, sodium cacodylate buffer, *I*=0.05 M.

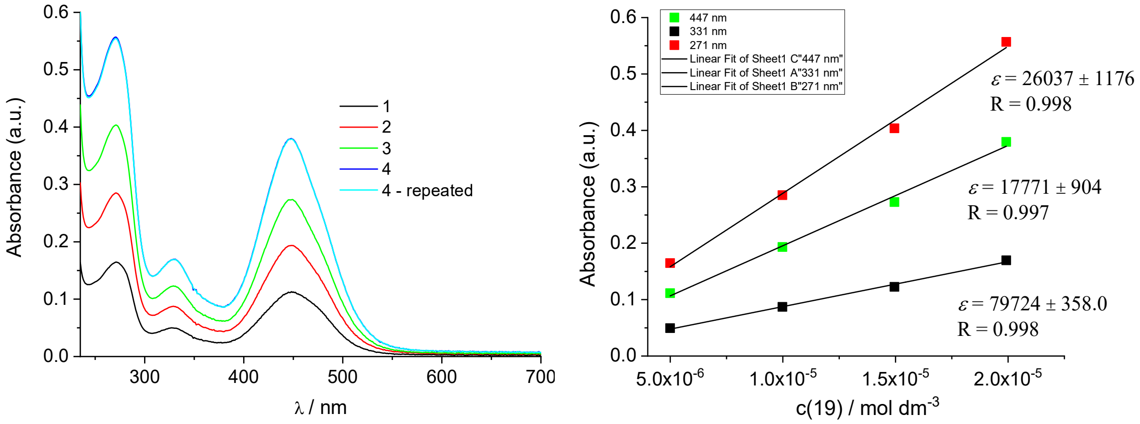

Figure S2. UV/Vis spectra changes of **19** at different concentrations (concentration range from 5.0×10^{-6} to 1.99×10^{-5} mol dm^{-3}) at pH=7.0, sodium cacodylate buffer, *I*=0.05 M.

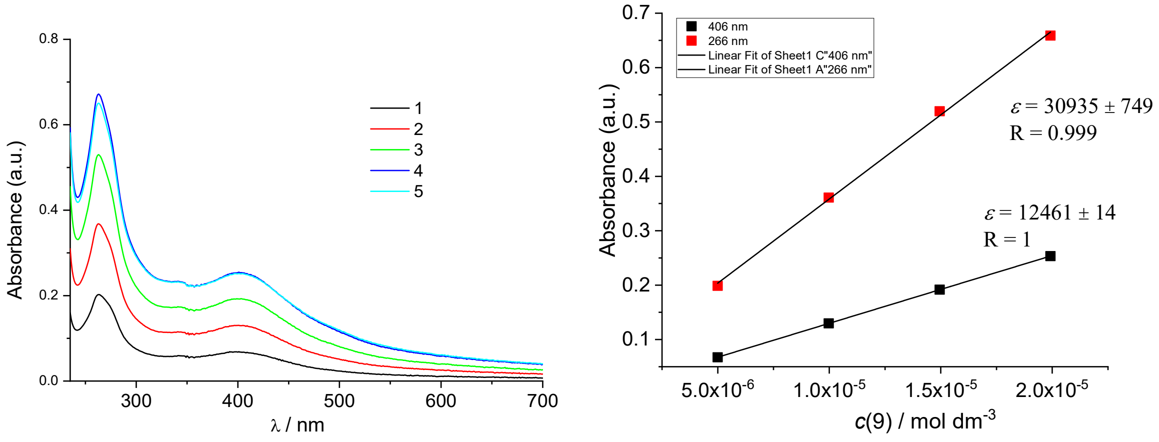

Figure S3. UV/Vis spectra changes of **9** at different concentrations (concentration range from 5.0×10^{-6} to 1.99×10^{-5} mol dm^{-3}) at pH=7.0, sodium cacodylate buffer, *I*=0.05 M.

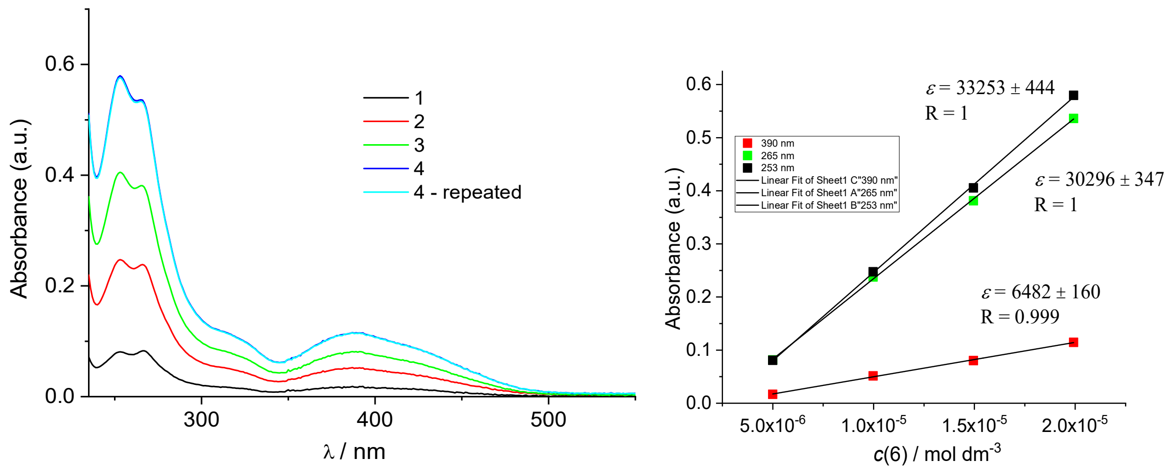

Figure S4. UV/Vis spectra changes of **6** at different concentrations (concentration range from 5.0×10^{-6} to 1.99×10^{-5} mol dm^{-3}) at pH=7.0, sodium cacodylate buffer, I=0.05 M.

Table S1. Electronic absorption data of **6, 9, 19** and **22**.

	pH = 7.0[a]	
	λ_{max} / nm	$\varepsilon \times 10^3$ / $mmol^{-1}$ cm^2
**6**	253	33.25
	265	30.30
	390	6.48
**9**	266	30.93
	406	12.46
**19**	271	26.04
	447	17.77
**22**	268	21.48
	436	13.13

[a] Sodium cacodylate buffer, I = 0.05 mol dm^{-3}, pH = 7.0.

The emission intensities of buffered aqueous solutions (sodium cacodylate buffer, I = 0.05 mol dm^{-3}, pH = 7.0) of studied compounds were proportional to their concentrations up to c = 2.0×10^{-6} mol dm^{-3} (Figures S5-S8).

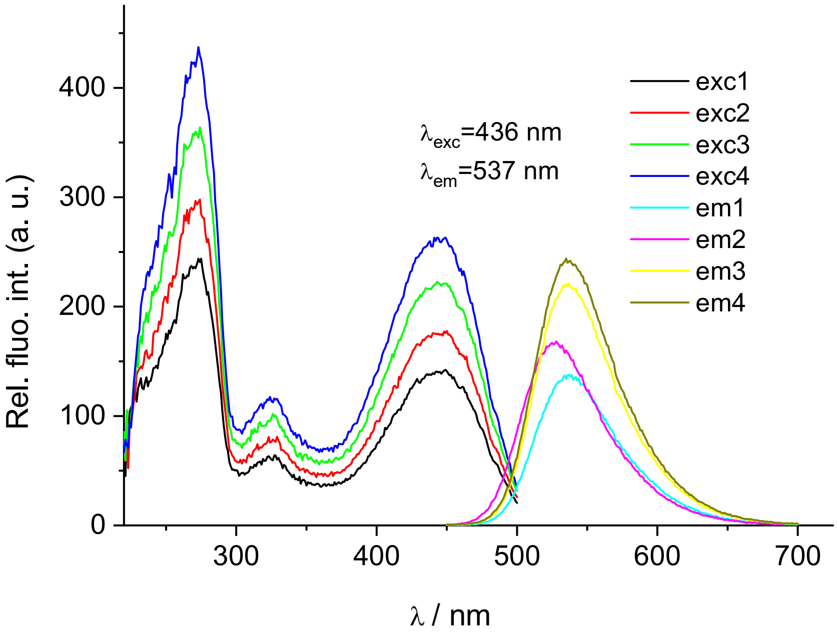

Figure S5. Emission and excitation spectra changes of **22** at different concentrations at λ_{exc}=436 nm (concentration range from 5.0×10^{-7} to 2.0×10^{-6} mol dm^{-3}) at pH=7.0, Na cacodylate buffer, I=0.05 mol dm^{-3}.

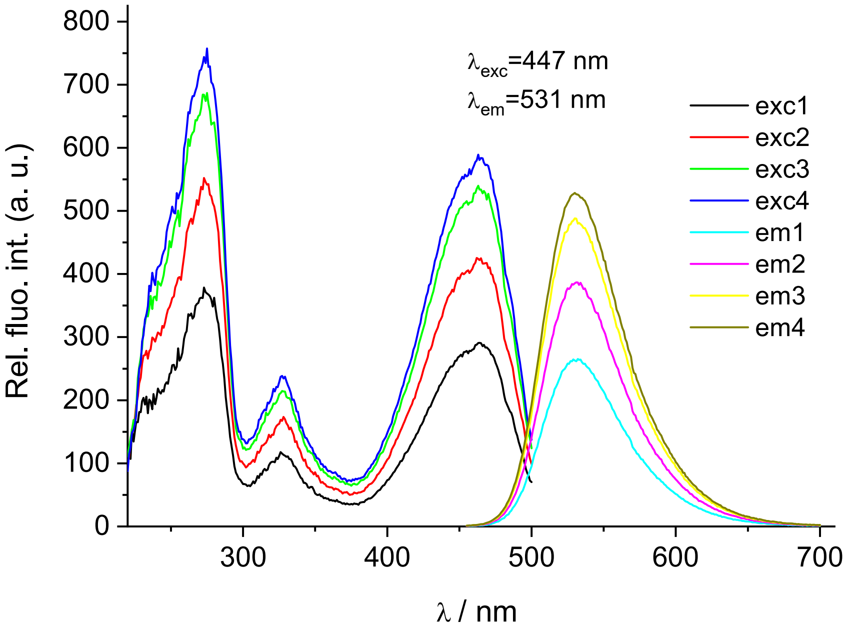

Figure S6. Emission and excitation spectra changes of **19** at different concentrations at λ_{exc}=447 nm (concentration range from 5.0×10^{-7} to 2.0×10^{-6} mol dm^{-3}) at pH=7.0, Na cacodylate buffer, I=0.05 mol dm^{-3}.

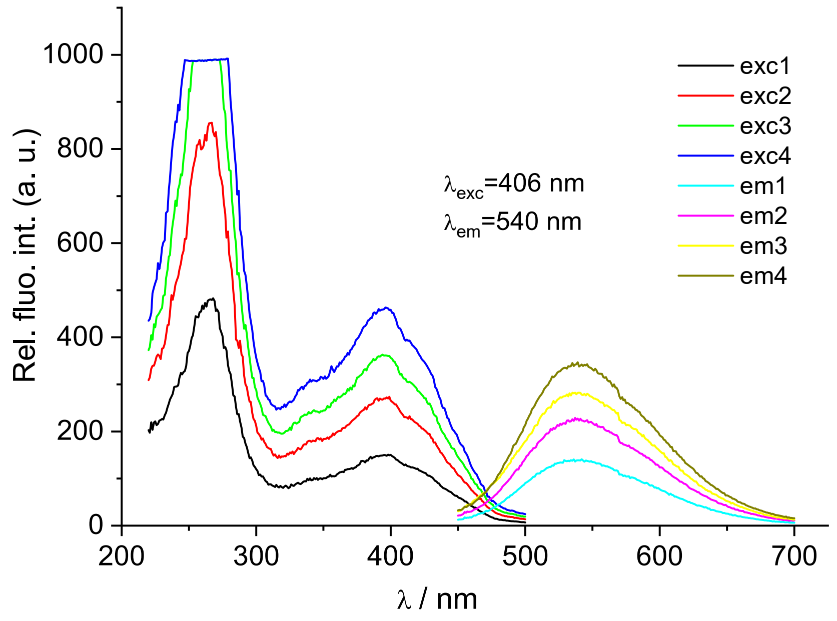

Figure S7. Emission and excitation spectra changes of **9** at different concentrations at λ_{exc}=406 nm (concentration range from 5.0 × 10^{-7} to 2.0 × 10^{-6} mol dm^{-3}) at pH=7.0, Na cacodylate buffer, *I*=0.05 mol dm^{-3}.

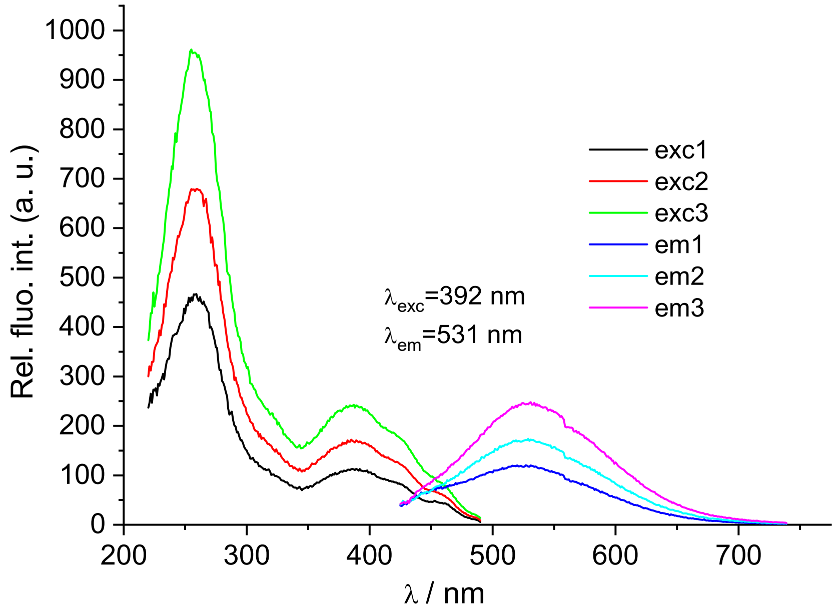

Figure S8. Emission and excitation spectra changes of **6** at different concentrations at λ_{exc}=392 nm (concentration range from 5.0 × 10^{-7} to 2.0 × 10^{-6} mol dm^{-3}) at pH=7.0, Na cacodylate buffer, *I*=0.05 mol dm^{-3}.

2. Interactions of pentacyclic benzimidazoles with ds-polynucleotides in neutral medium (pH=7.0)

2.1. Fluorimetric titrations

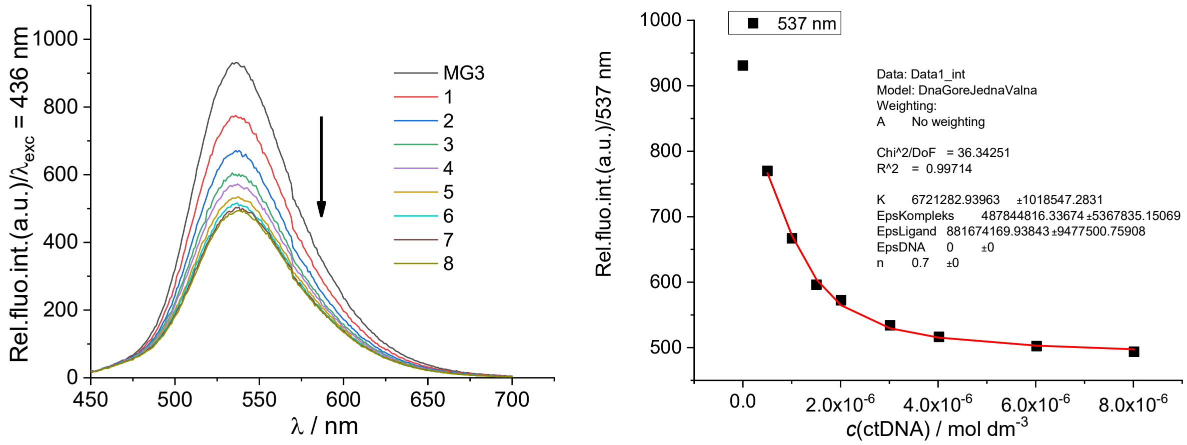

Figure S9. a) Changes in fluorescence spectrum of **22** (c= 1.0 × 10^{-6} mol dm^{-3}, λ_{exc}=436 nm) upon titration with ctDNA (c= 5.0 × 10^{-7} – 8.0 × 10^{-6} mol dm^{-3}); b) Dependence of **22** absorbance at λ_{max} = 537 nm on c(ctDNA), at pH=7.0, sodium cacodylate buffer, I = 0.05 mol dm^{-3}.

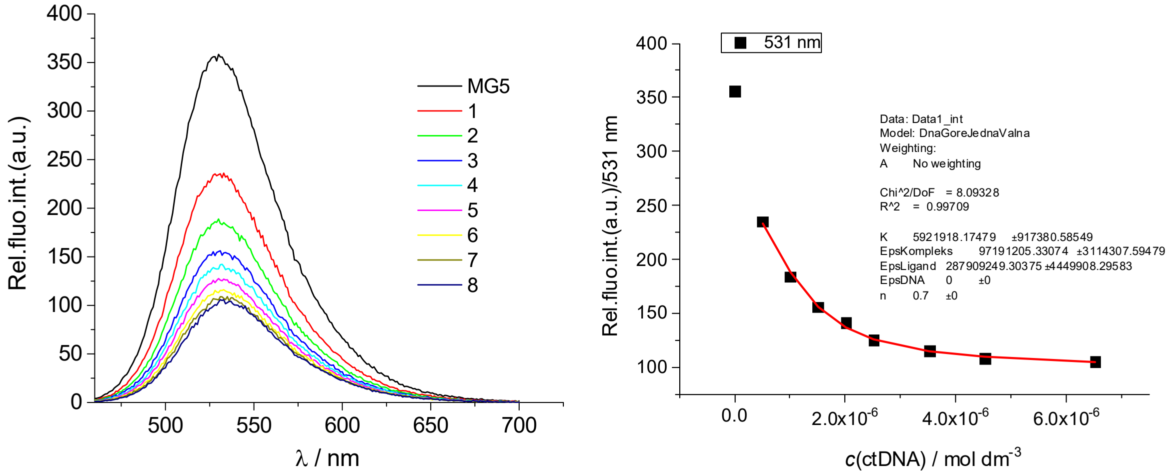

Figure S10. a) Changes in fluorescence spectrum of **19** (c= 1.0 × 10^{-6} mol dm^{-3}, λ_{exc}=447 nm) upon titration with ctDNA (c= 5.0 × 10^{-7} – 6.5 × 10^{-6} mol dm^{-3}); b) Dependence of **19** absorbance at λ_{max} = 531 nm on c(ctDNA), at pH=7.0, sodium cacodylate buffer, I = 0.05 mol dm^{-3}.

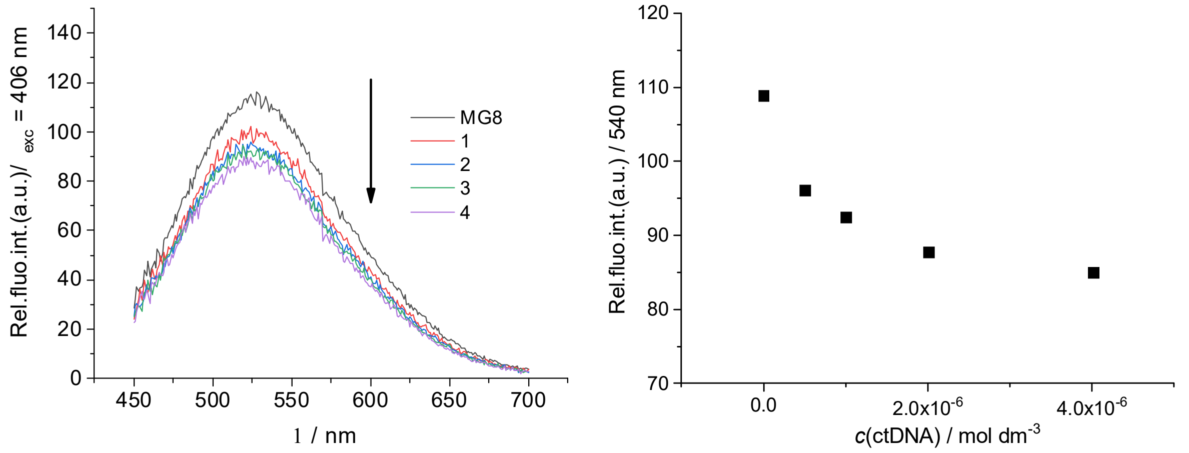

Figure S11. a) Changes in fluorescence spectrum of **9** (c= 1.0 × 10^{-6} mol dm^{-3}, λ_{exc}=406 nm) upon titration with ctDNA (c= 5.0 × 10^{-7} – 4.0 × 10^{-6} mol dm^{-3}); b) Dependence of **9** absorbance at λ_{max} = 540 nm on c(ctDNA), at pH=7.0, sodium cacodylate buffer, I = 0.05 mol dm^{-3}.

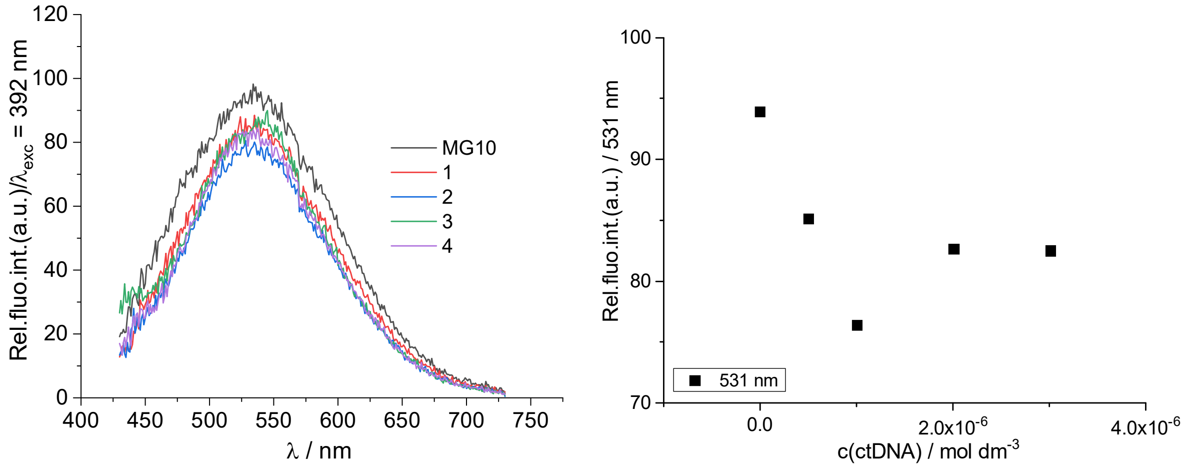

Figure S12. a) Changes in fluorescence spectrum of **6** (c= 1.0 × 10^{-6} mol dm^{-3}, λ_{exc}=392 nm) upon titration with ctDNA (c= 5.0 × 10^{-7} – 4.0 × 10^{-6} mol dm^{-3}); b) Dependence of **6** absorbance at λ_{max} = 531 nm on c(ctDNA), at pH=7.0, sodium cacodylate buffer, I = 0.05 mol dm^{-3}.

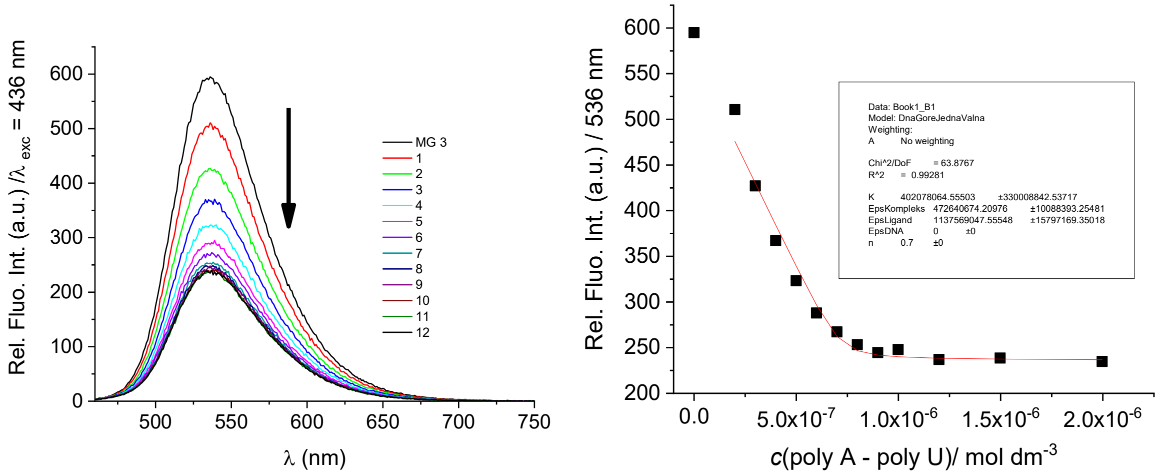

Figure S13. a) Changes in fluorescence spectrum of **22** (c= 5.0 × 10^{-7} mol dm^{-3}, λ_{exc}=436 nm) upon titration with rArU (c= 2.0 × 10^{-7} – 2.0 × 10^{-6} mol dm^{-3}); b) Dependence of **22** absorbance at λ_{max} = 536 nm on c(rArU), at pH=7.0, sodium cacodylate buffer, I = 0.05 mol dm^{-3}.

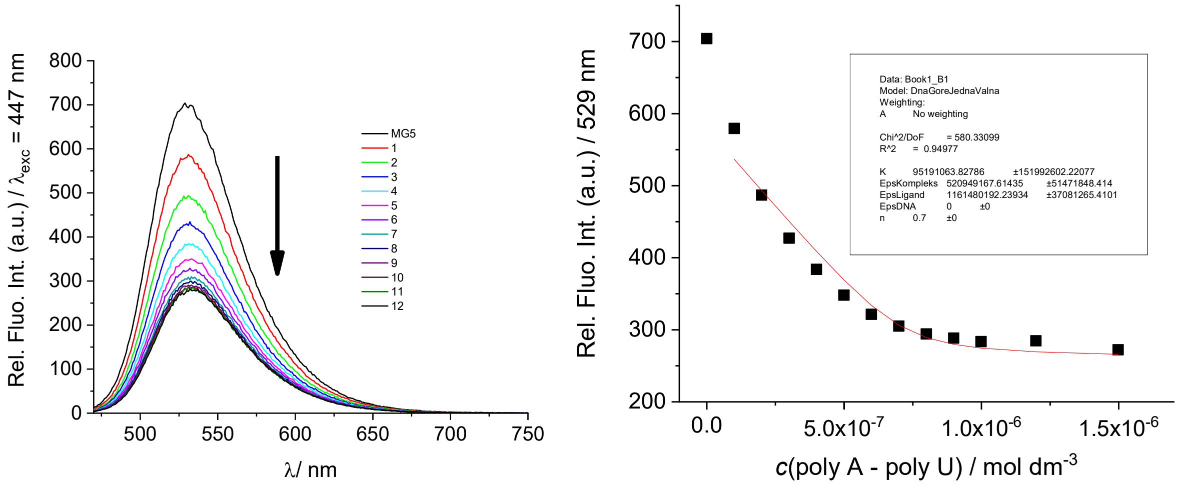

Figure S14. a) Changes in fluorescence spectrum of **19** (c= 5.0 × 10^{-7} mol dm^{-3}, λ_{exc}=447 nm) upon titration with rArU (c= 1.0 × 10^{-7} – 1.5 × 10^{-6} mol dm^{-3}); b) Dependence of **19** absorbance at λ_{max} = 529 nm on c(rArU), at pH=7.0, sodium cacodylate buffer, I = 0.05 mol dm^{-3}.

2.2. Thermal melting experiments

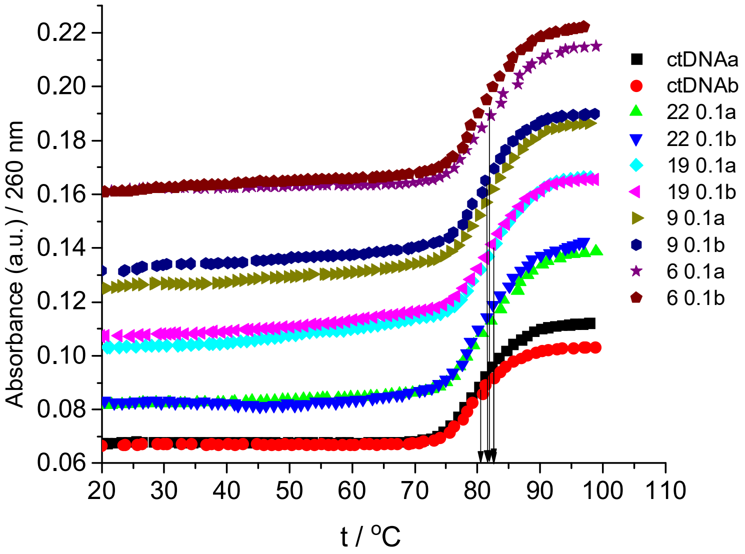

Figure S15. Melting curve of **ctDNA** upon addition of ratio, ***r*** ([compound/ [polynucleotide])=0.1 of **6, 9, 19** and **22** at pH = 7.0 (buffer sodium cacodylate, I = 0.05 mol dm^{-3}).

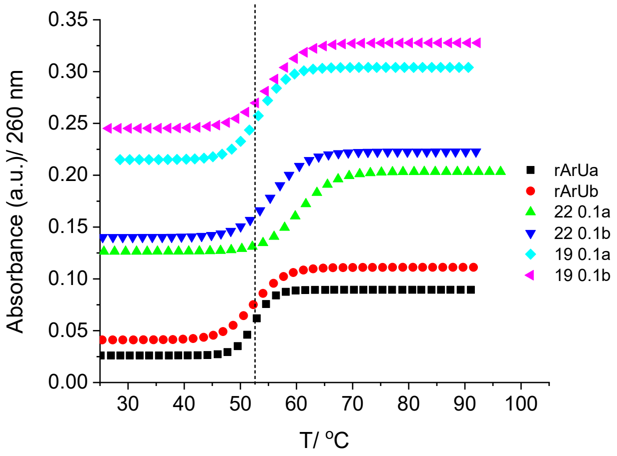

Figure S16. Melting curve of **rArU** upon addition of ratio, ***r*** ([compound/ [polynucleotide]) = 0.1 of **19** and **22** at pH = 7.0 (buffer sodium cacodylate, $I = 0.05$ mol dm^{-3}).

2.3. Circular dichroism (CD) titrations

	**22**	**19**	**9**	**6**
**ct DNA**	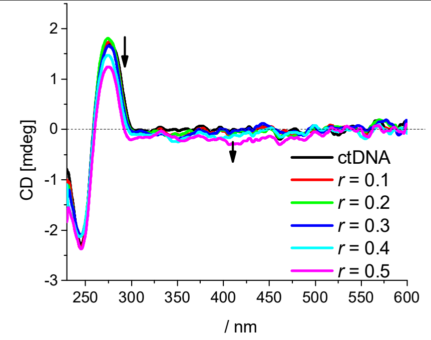	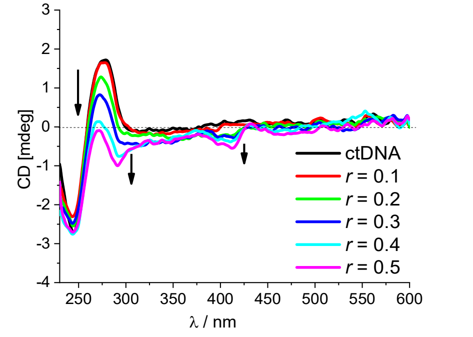	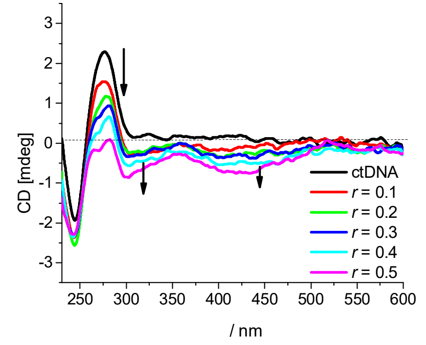	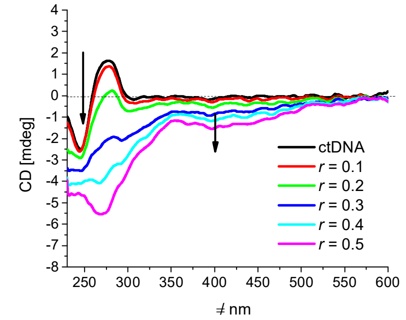
**rArU**	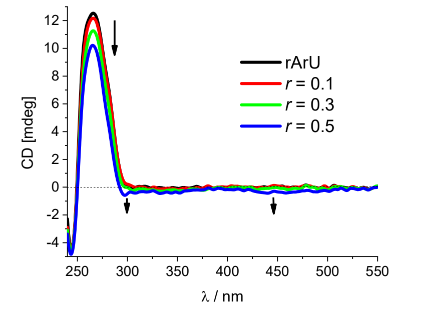	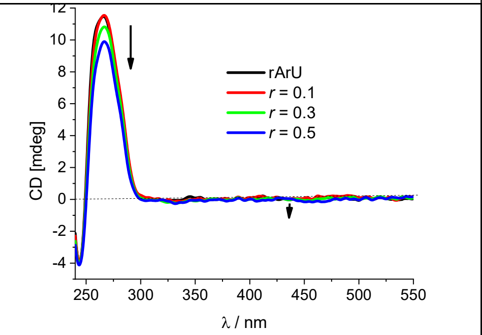		

Figure S17. CD titration of ctDNA and rArU (c = 3.0 × 10^{-5} mol dm^{-3}) with **6, 9, 19** and **22** at molar ratios **r** = [compound] / [polynucleotide] (pH = 7.0, buffer sodium cacodylate, I = 0.05 mol dm^{-3}).

3. NMR spectra of synthesized compounds

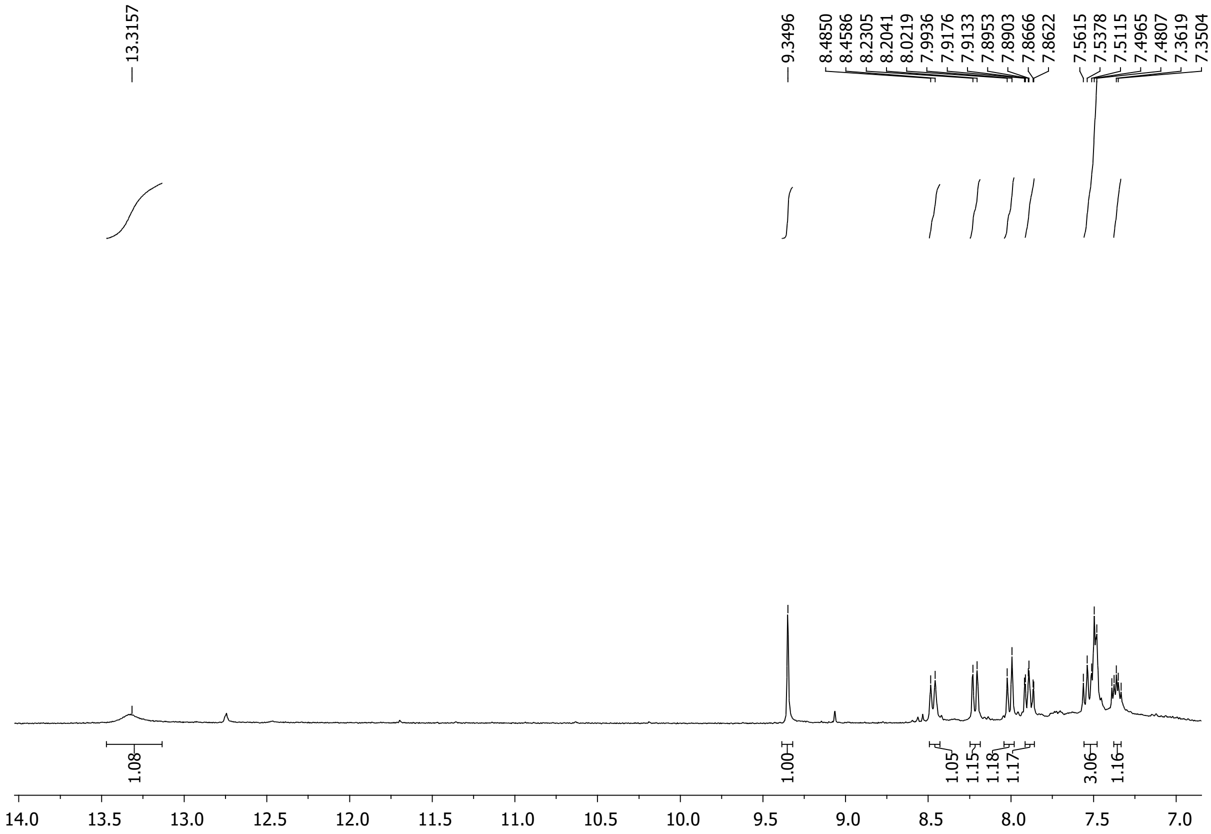

Figure S18. ^{1}H NMR spectrum (DMSO-d_6, 300 MHz) of *7-oxo-5,7-dihydrobenzo[g]benzo[4,5]imidazo[1,2-a][1,8]naphthyridine-6-carbonitrile* ***4***

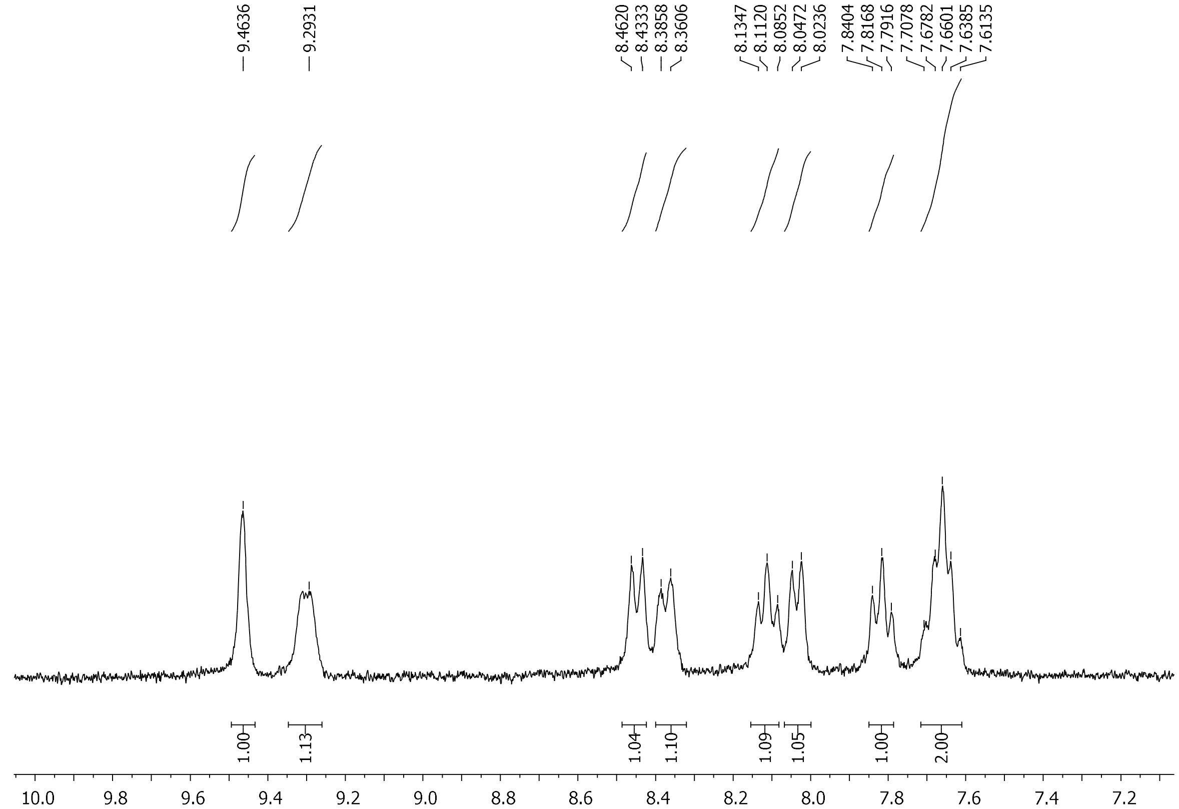

Figure S19. ^{1}H NMR spectrum (DMSO-d_6, 300 MHz) of *7-chlorobenzo[g]benzo[4,5]imidazo[1,2-a][1,8]naphthyridine-6-carbonitrile* ***5***

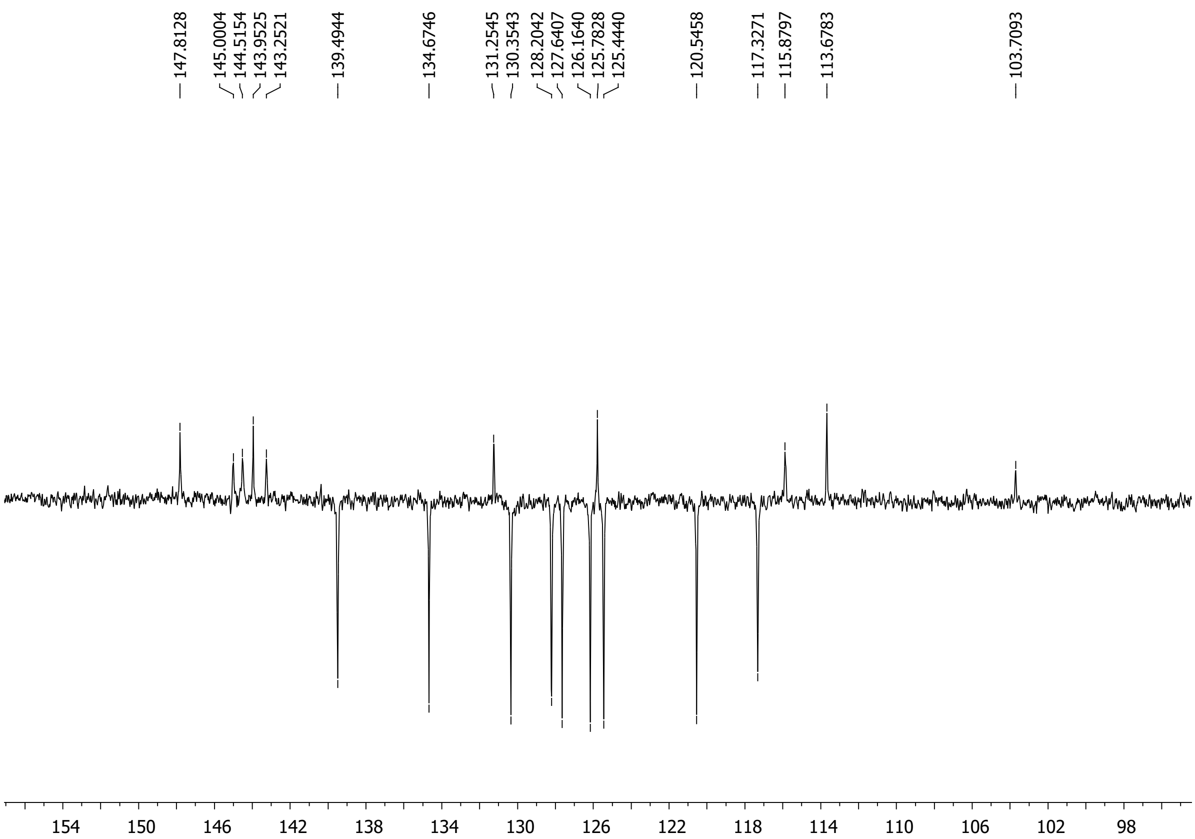

Figure S20. ^{13}C NMR spectrum (DMSO-d_6, 100 MHz) of *7-chlorobenzo[g]benzo[4,5]imidazo[1,2-a][1,8]naphthyridine-6-carbonitrile* ***5***

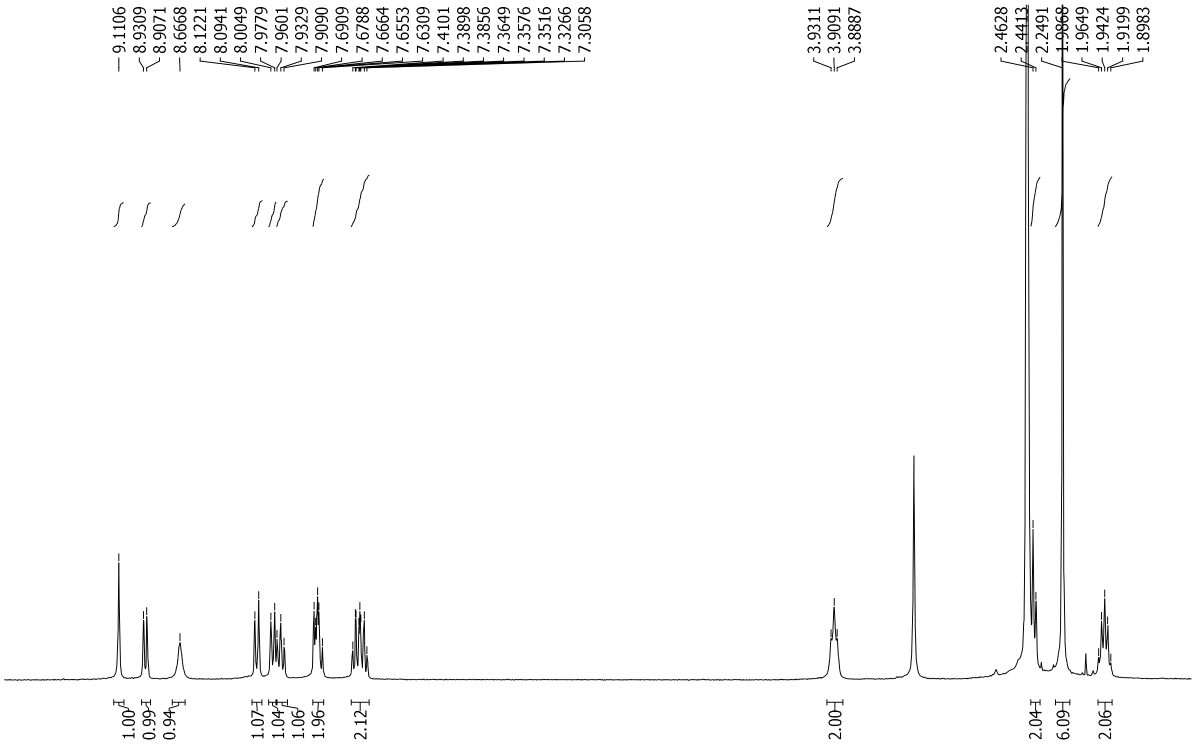

Figure S21. ^{1}H NMR spectrum (DMSO-d_6, 300 MHz) of *7-((3-N,N-(dimethylamino)propyl)amino)benzo[g]benzo[4,5]imidazo[1,2-a][1,8]naphthyridine-6-carbonitrile* ***6***

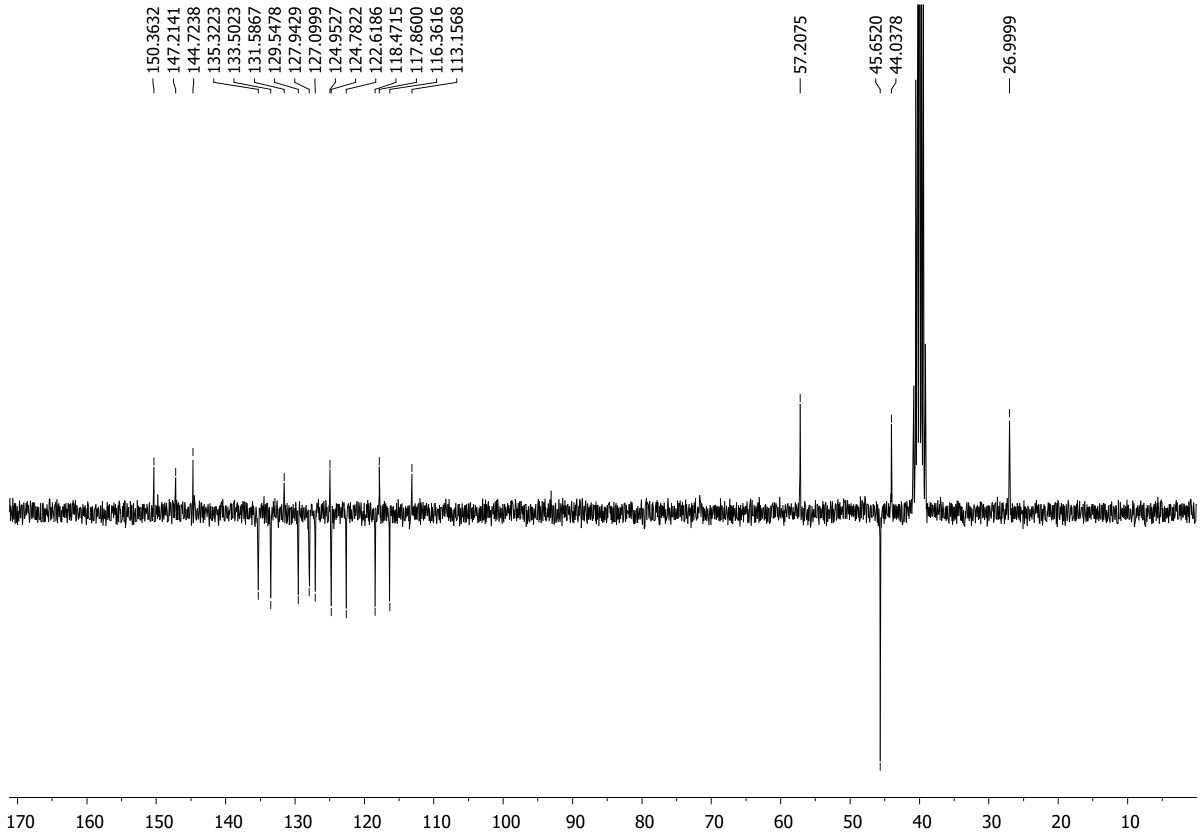

Figure S22. ^{13}C NMR spectrum (DMSO-d_6, 75 MHz) of *7-((3-N,N-(dimethylamino)propyl)amino)benzo[g]benzo[4,5]imidazo[1,2-a][1,8]naphthyridine-6-carbonitrile* ***6***

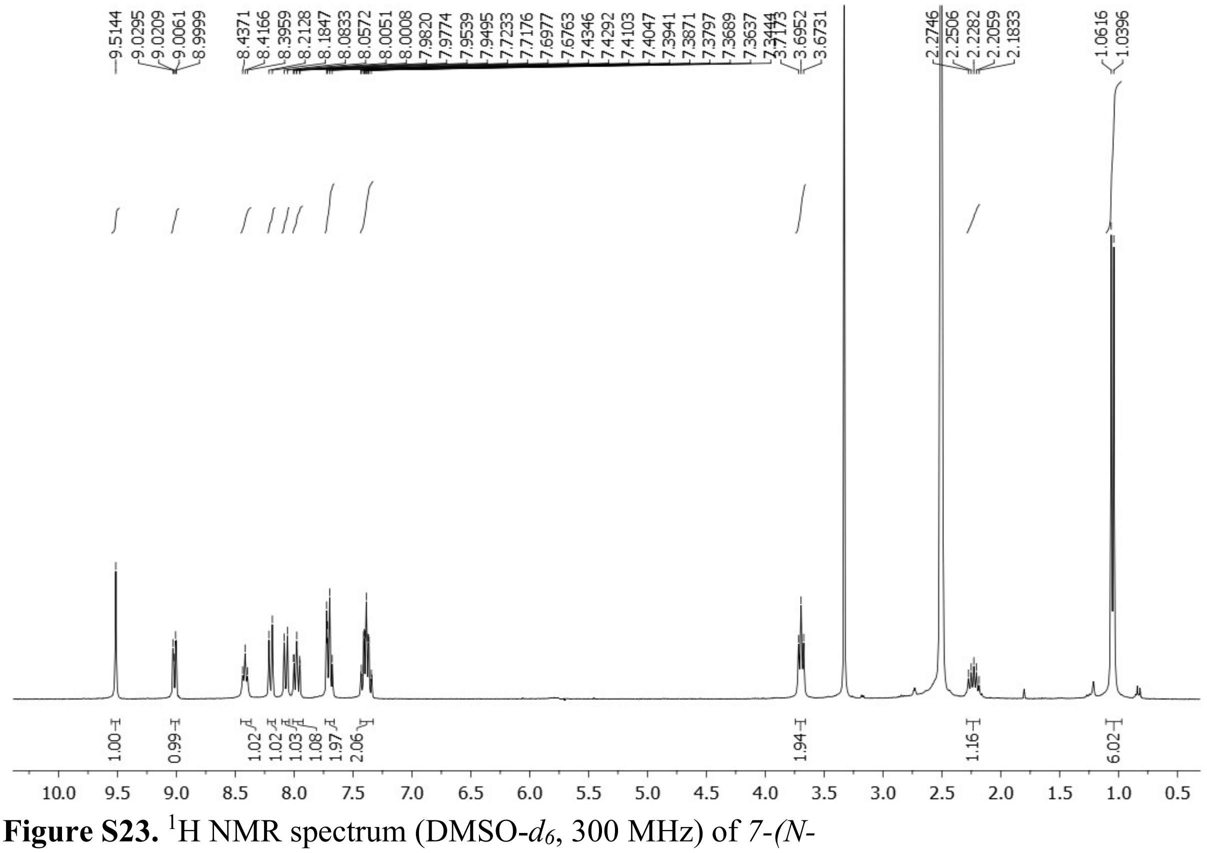

Figure S23. ^{1}H NMR spectrum (DMSO-d_6, 300 MHz) of *7-(N-isobutylamino)benzo[g]benzo[4,5]imidazo[1,2-a][1,8]naphthyridine-6-carbonitrile* ***7***

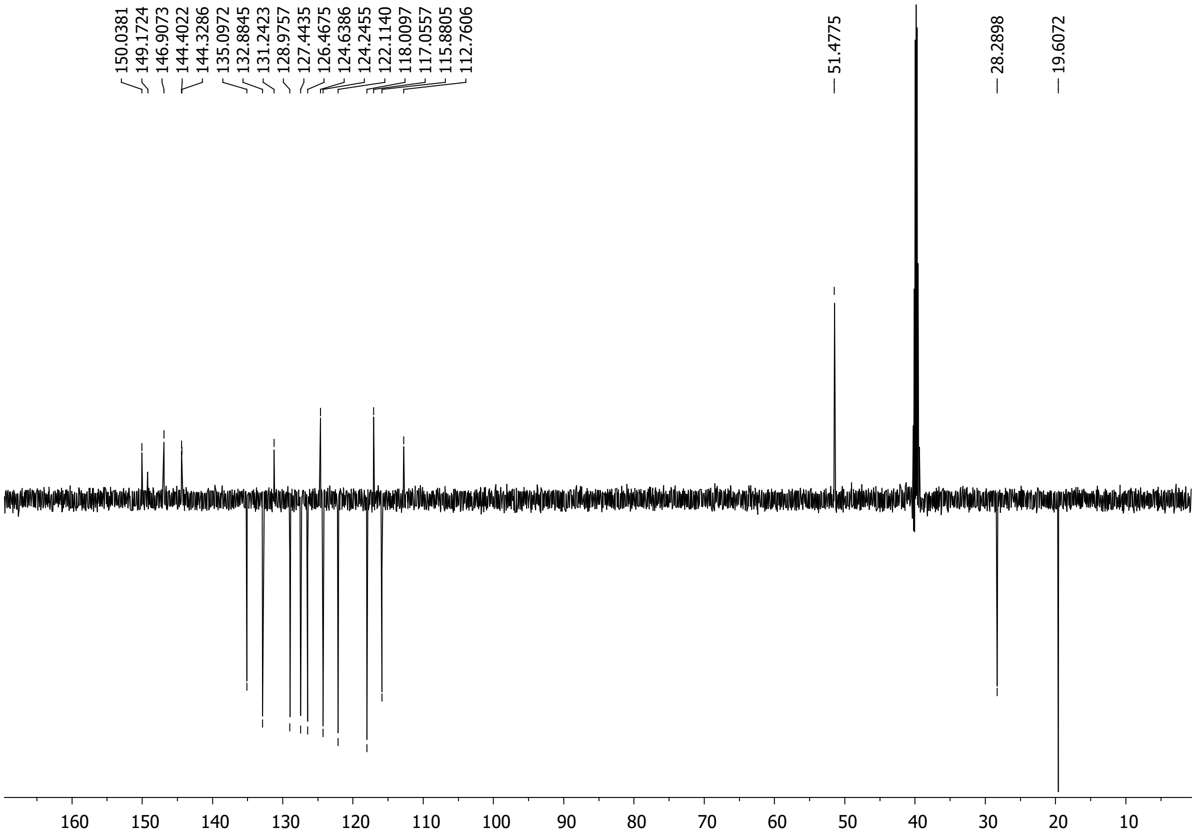

Figure S24. ^{13}C NMR spectrum (DMSO-d_6, 75 MHz) of *7-(N-isobutylamino)benzo[g]benzo[4,5]imidazo[1,2-a][1,8]naphthyridine-6-carbonitrile* ***7***

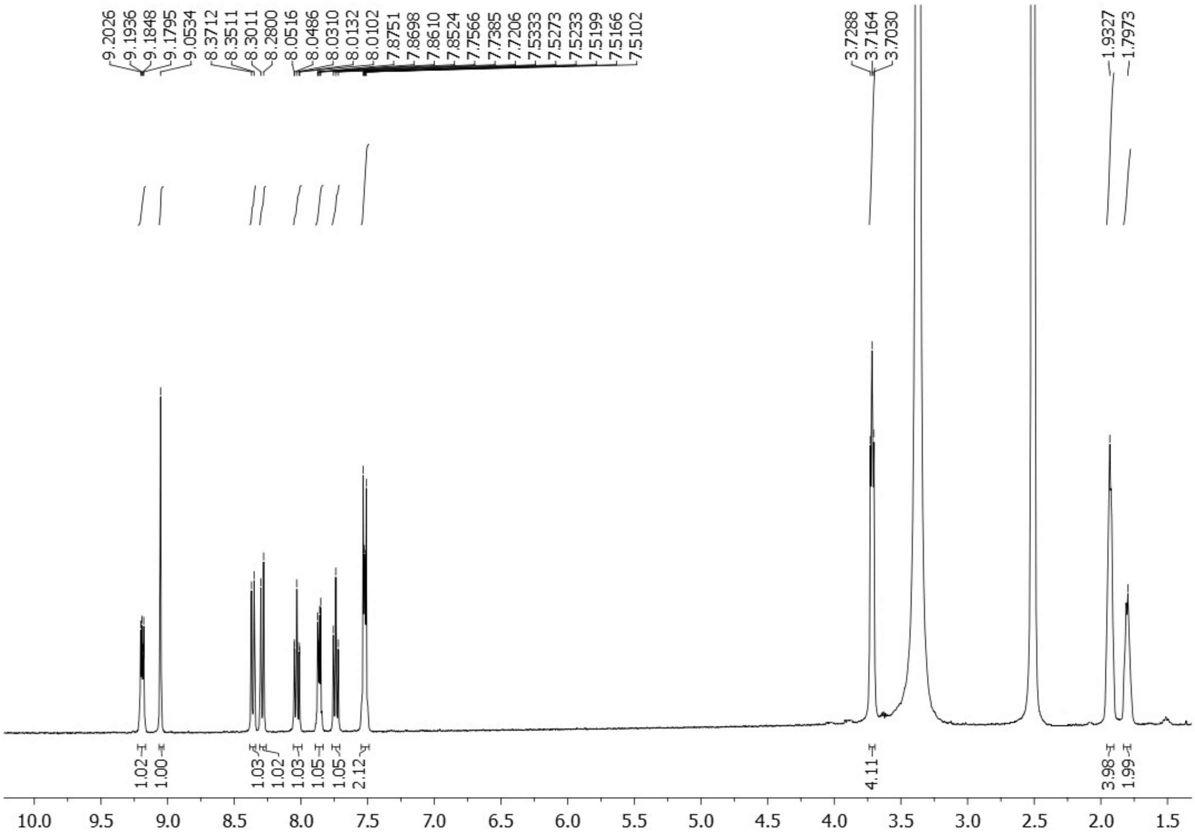

Figure S25. ^{1}H NMR spectrum (DMSO-d_6, 300 MHz) of *7-(piperidin-1-yl)benzo[g]benzo[4,5]imidazo[1,2-a][1,8]naphthyridine-6-carbonitrile* ***8***

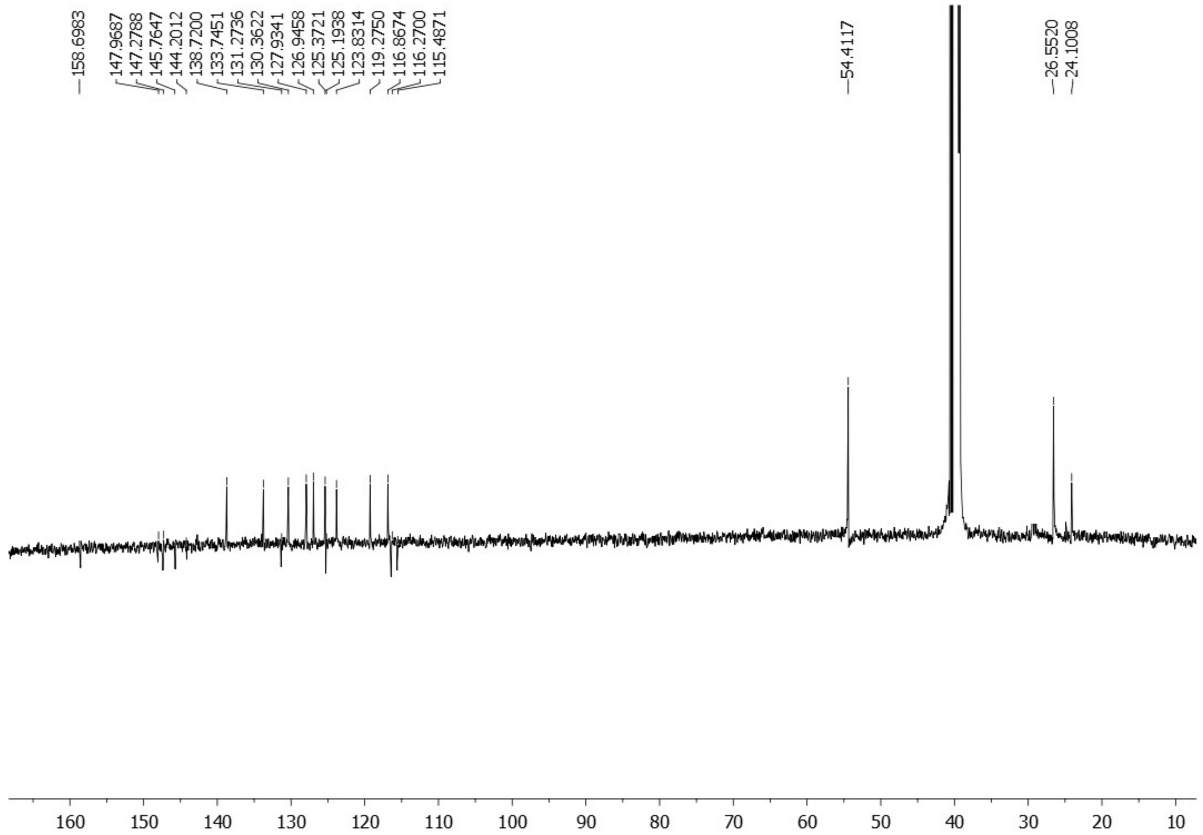

Figure S26. ^{13}C NMR spectrum (DMSO-d_6, 75 MHz) of *7-(piperidin-1-yl)benzo[g]benzo[4,5]imidazo[1,2-a][1,8]naphthyridine-6-carbonitrile* ***8***

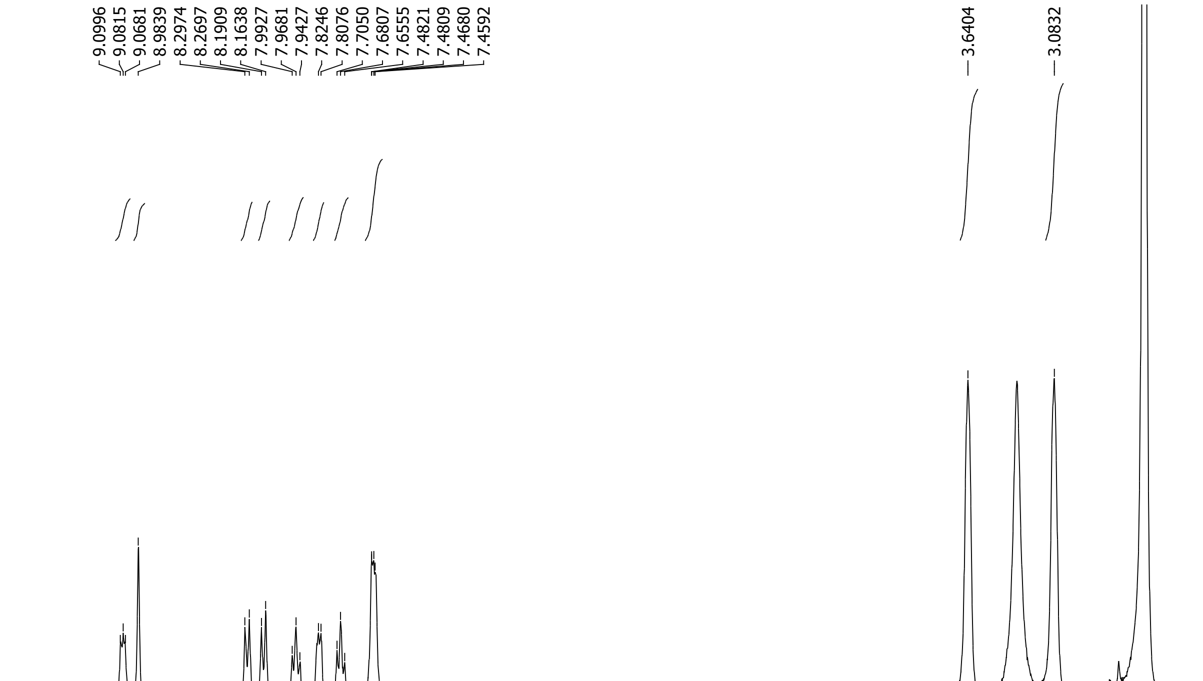

Figure S27. ^{1}H NMR spectrum (DMSO-d_6, 300 MHz) of *7-(piperazin-1-yl)benzo[g]benzo[4,5]imidazo[1,2-a][1,8]naphthyridine-6-carbonitrile* ***9***

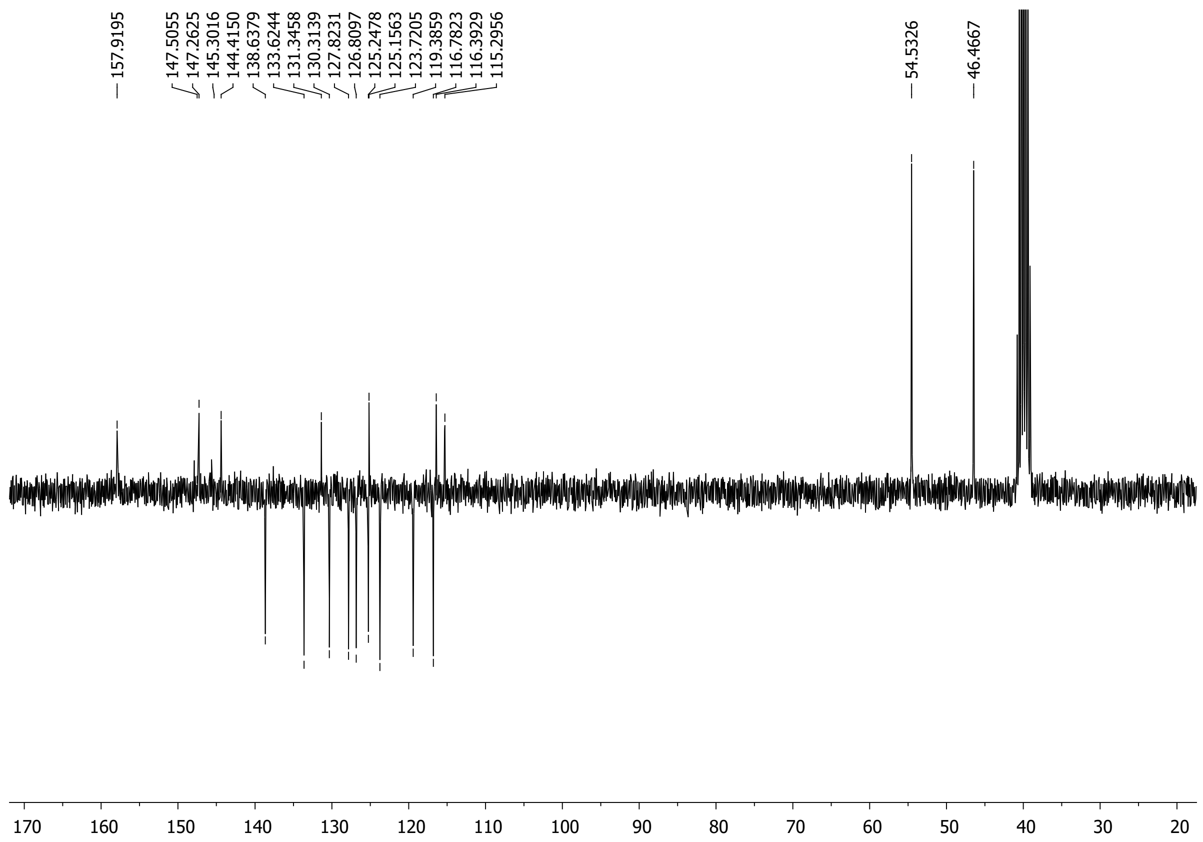

Figure S28. ^{13}C NMR spectrum (DMSO-d_6, 75 MHz) of *7-(piperazin-1-yl)benzo[g]benzo[4,5]imidazo[1,2-a][1,8]naphthyridine-6-carbonitrile* ***9***

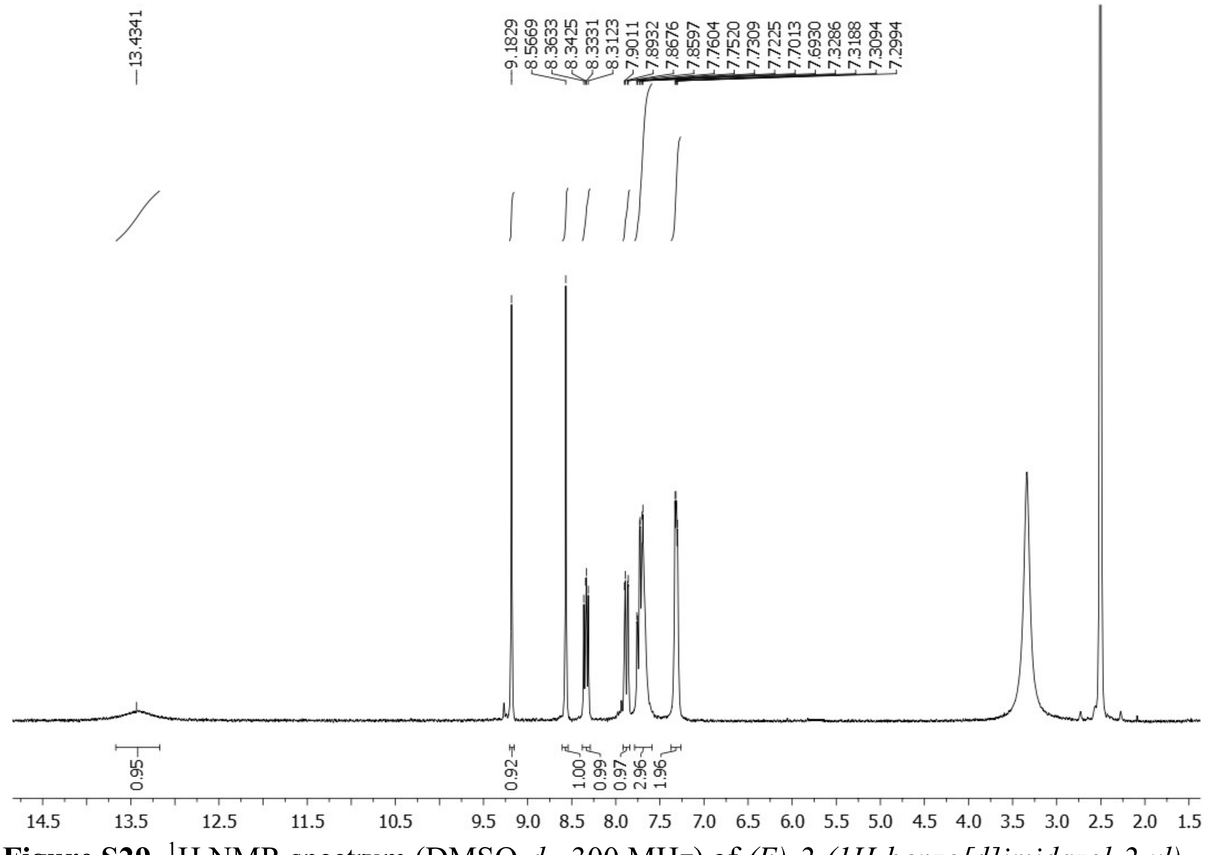

Figure S29. ^{1}H NMR spectrum (DMSO-d_6, 300 MHz) of *(E)-2-(1H-benzo[d]imidazol-2-yl)-3-(2-chloro-7-fluoroquinolin-3-yl)acrylonitrile* ***13***

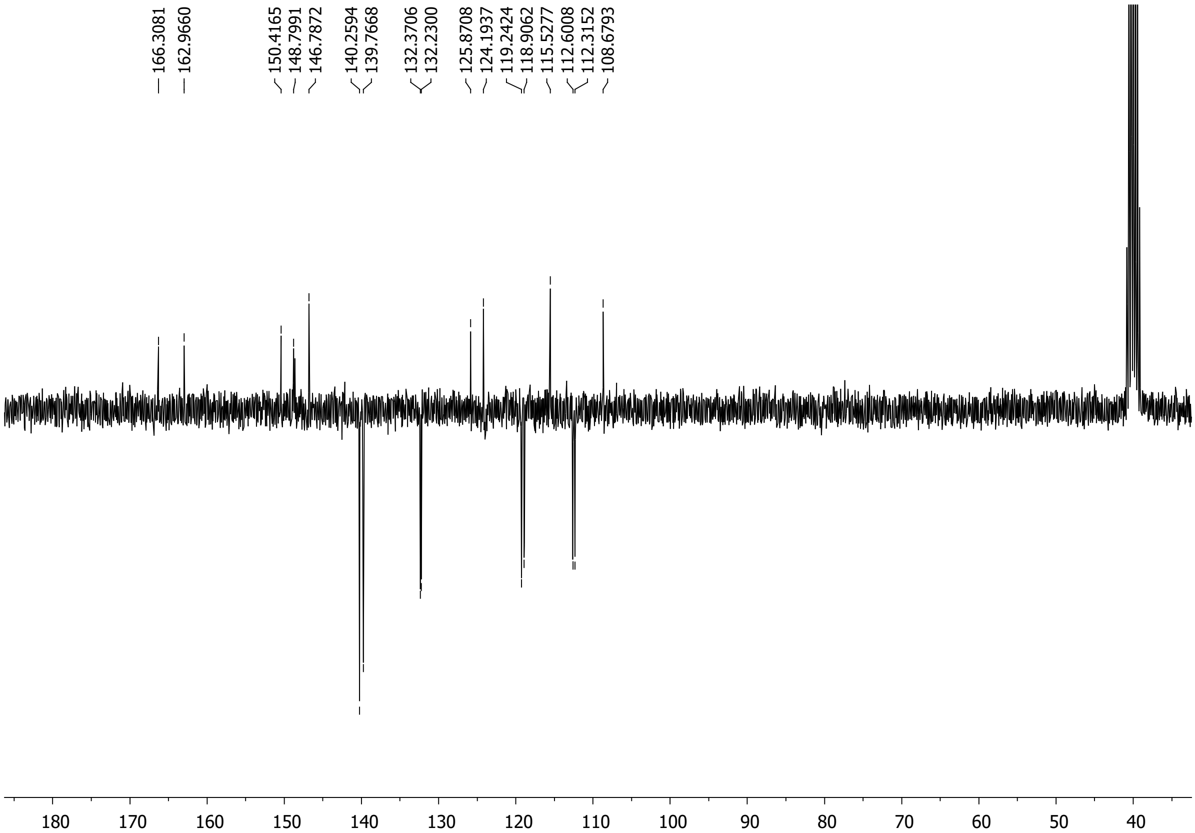

Figure S30. ^{13}C NMR spectrum (DMSO-d_6, 75 MHz) of *(E)-2-(1H-benzo[d]imidazol-2-yl)-3-(2-chloro-7-fluoroquinolin-3-yl)acrylonitrile* ***13***

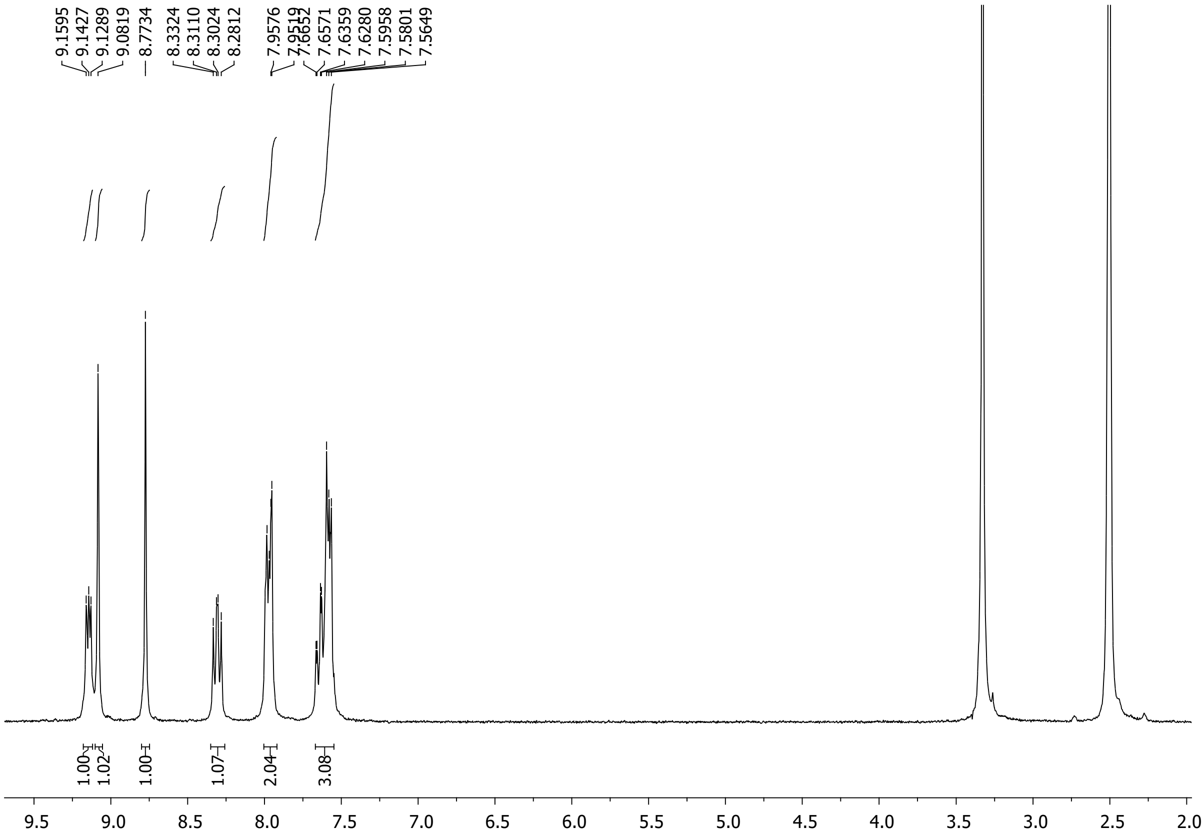

Figure S31. ^{1}H NMR spectrum (DMSO-d_6, 300 MHz) of *11-fluorobenzo[g]benzo[4,5]imidazo[1,2-a][1,8]naphthyridine-6-carbonitrile* ***16***

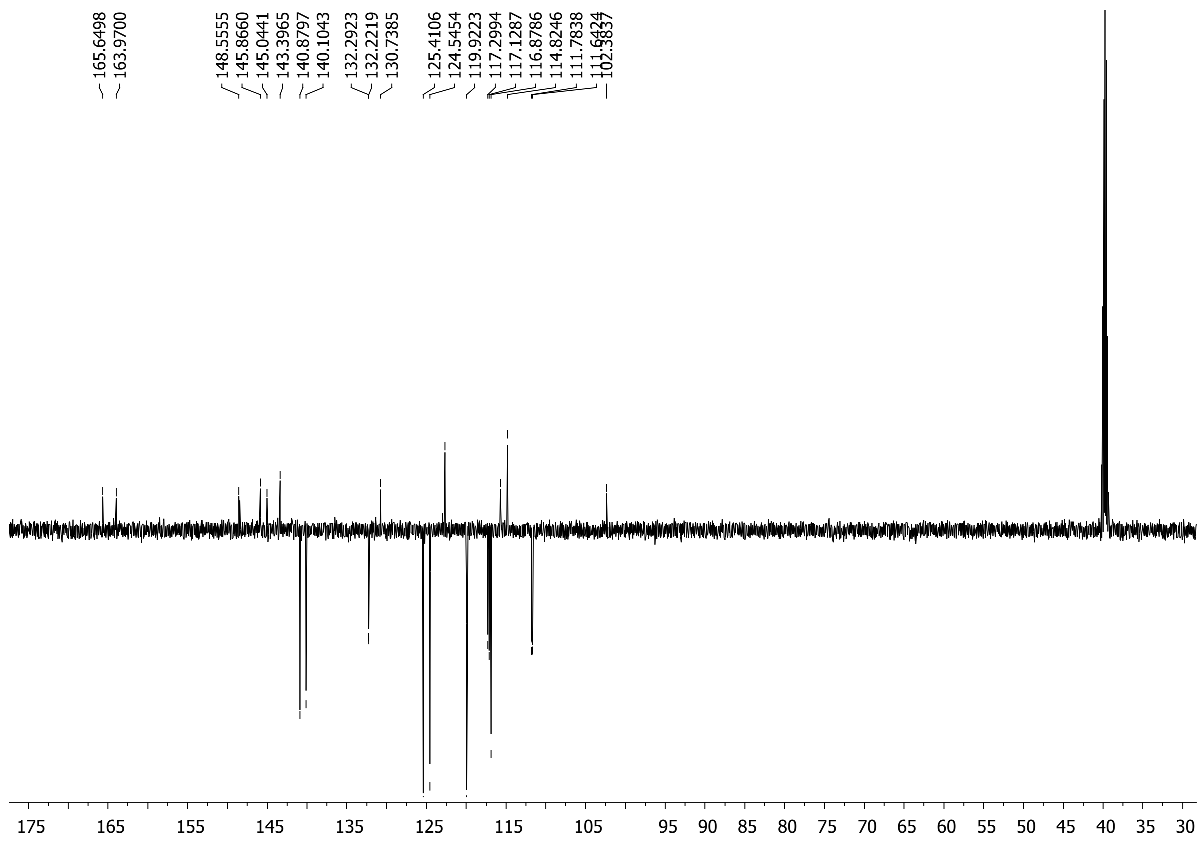

Figure S32. ^{13}C NMR spectrum (DMSO-d_6, 75 MHz) of *11-fluorobenzo[g]benzo[4,5]imidazo[1,2-a][1,8]naphthyridine-6-carbonitrile **16***

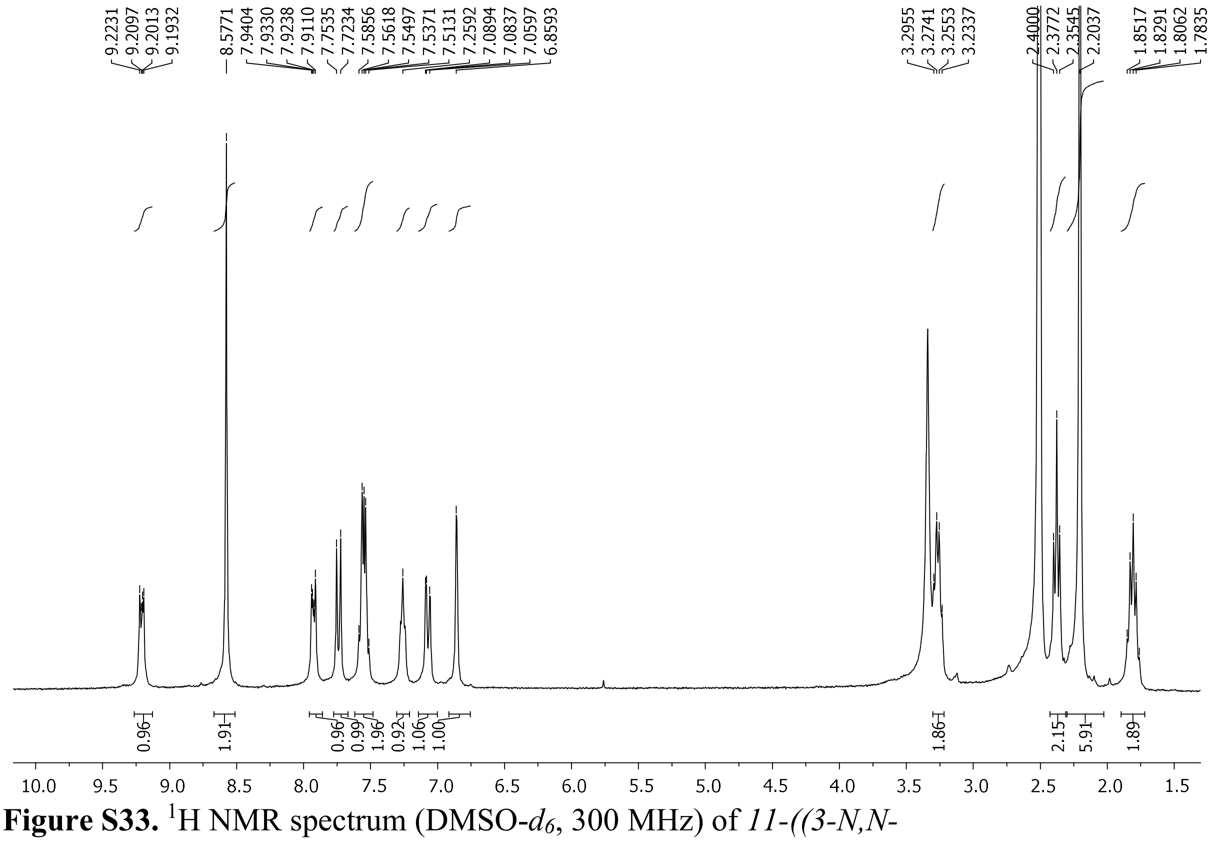

Figure S33. ^{1}H NMR spectrum (DMSO-d_6, 300 MHz) of *11-((3-N,N-(dimethylamino)propyl)amino)benzo[g]benzo[4,5]imidazo[1,2-a][1,8]naphthyridine-6-carbonitrile **19***

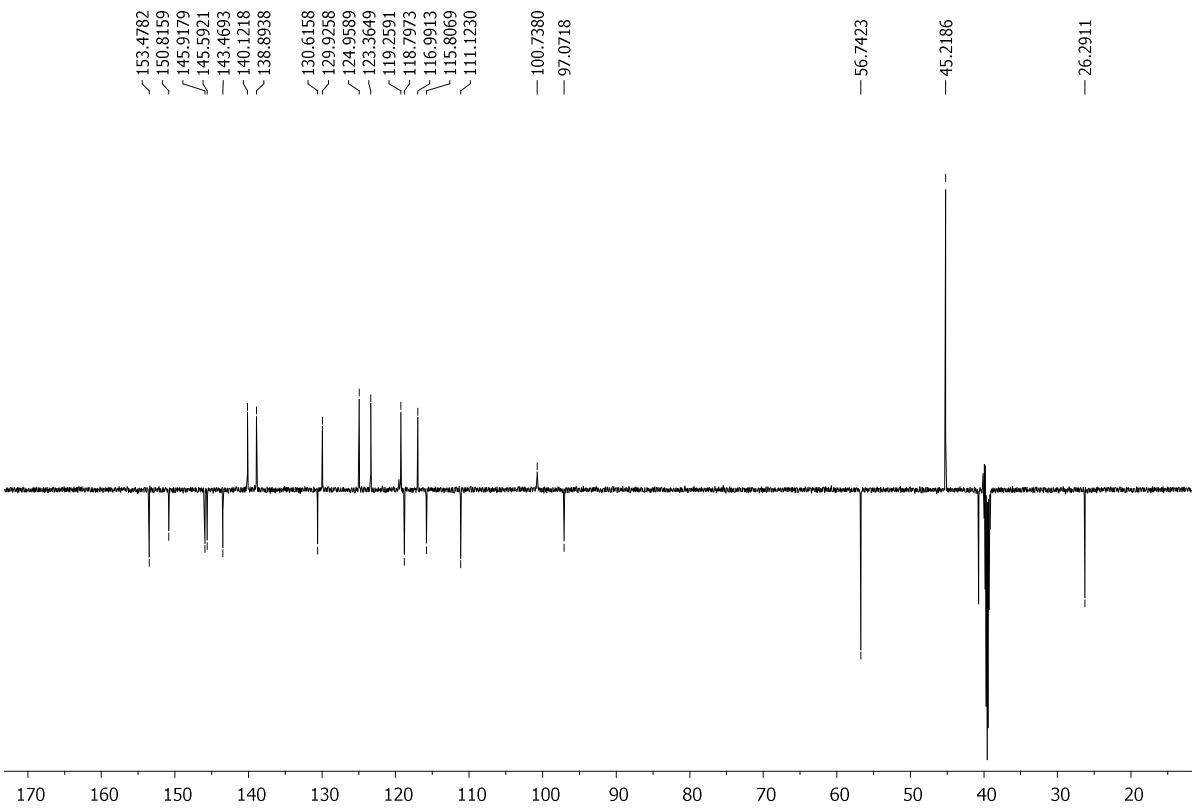

Figure S34. ^{13}C NMR spectrum (DMSO-d_6, 150 MHz) of *11-((3-N,N-(dimethylamino)propyl)amino)benzo[g]benzo[4,5]imidazo[1,2-a][1,8]naphthyridine-6-carbonitrile* ***19***

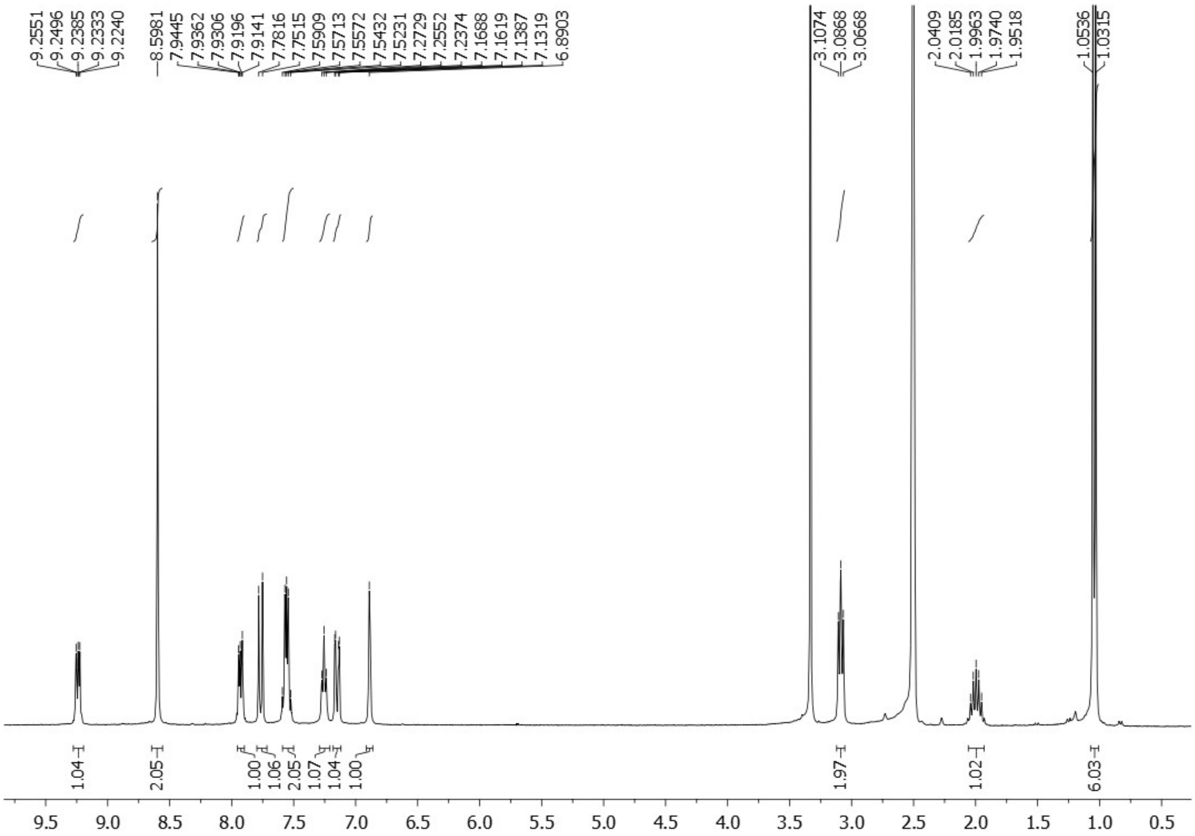

Figure S35. ^{1}H NMR spectrum (DMSO-d_6, 300 MHz) of *11-(N-isobutylamino)benzo[g]benzo[4,5]imidazo[1,2-a][1,8]naphthyridine-6-carbonitrile* ***20***

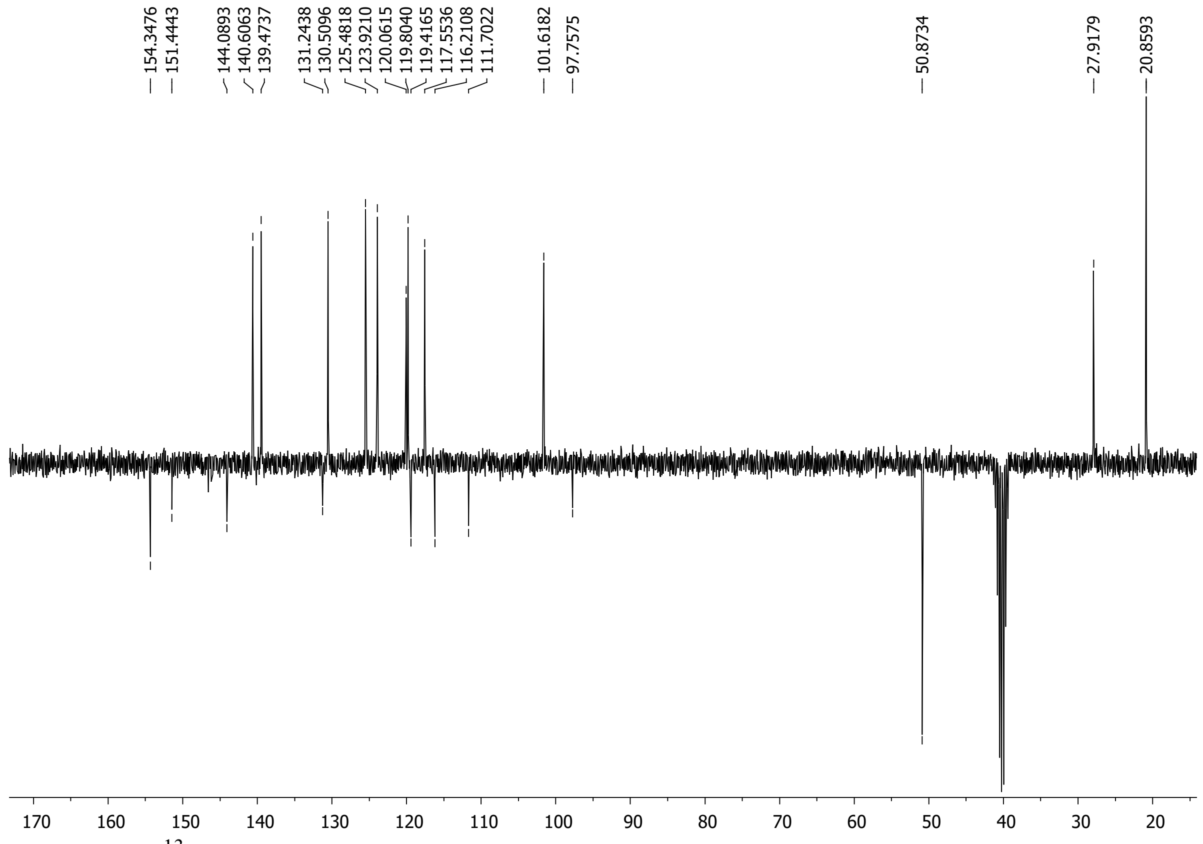

Figure S36. ^{13}C NMR spectrum (DMSO-d_6, 75 MHz) of *11-(N-isobutylamino)benzo[g]benzo[4,5]imidazo[1,2-a][1,8]naphthyridine-6-carbonitrile* ***20***

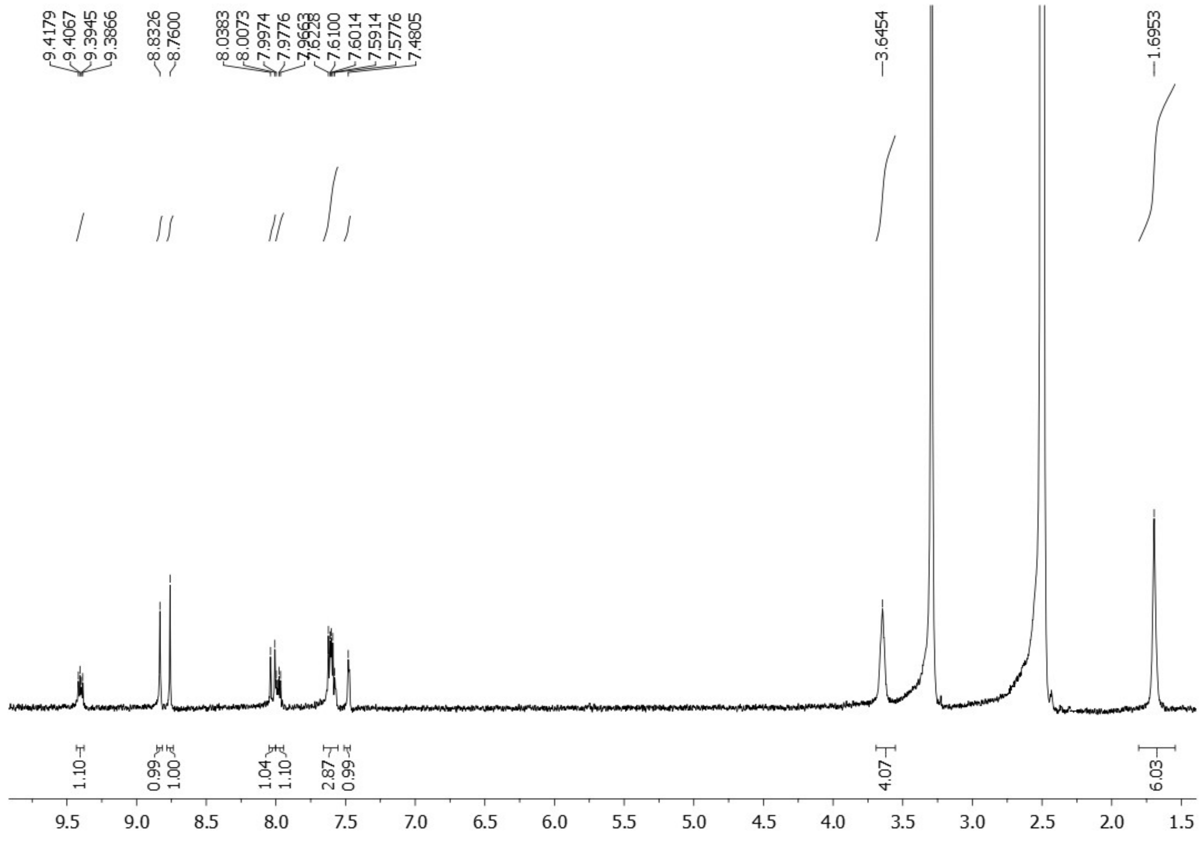

Figure S37. ^{1}H NMR spectrum (DMSO-d_6, 300 MHz) of *11-(piperidin-1-yl)benzo[g]benzo[4,5]imidazo[1,2-a][1,8]naphthyridine-6-carbonitrile* ***21***

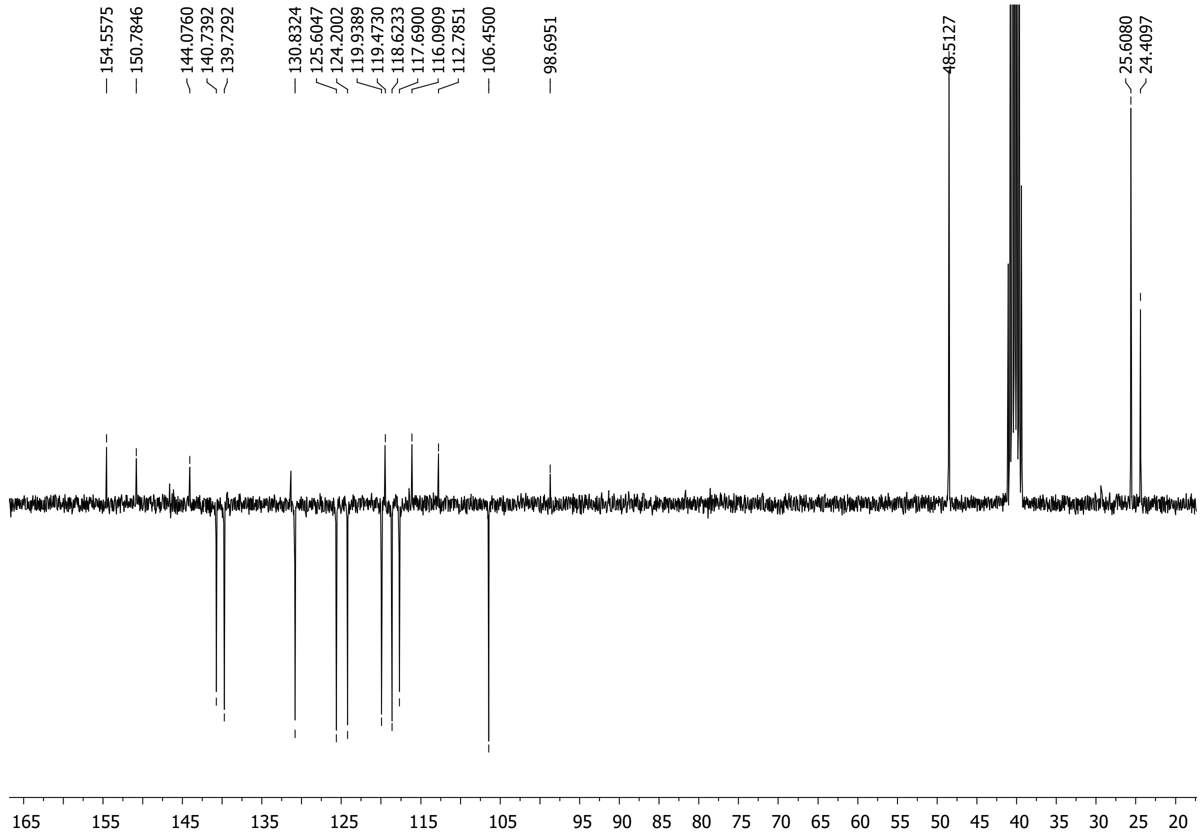

Figure S38. ^{13}C NMR spectrum (DMSO-d_6, 75 MHz) of *11-(piperidin-1-yl)benzo[g]benzo[4,5]imidazo[1,2-a][1,8]naphthyridine-6-carbonitrile **21***

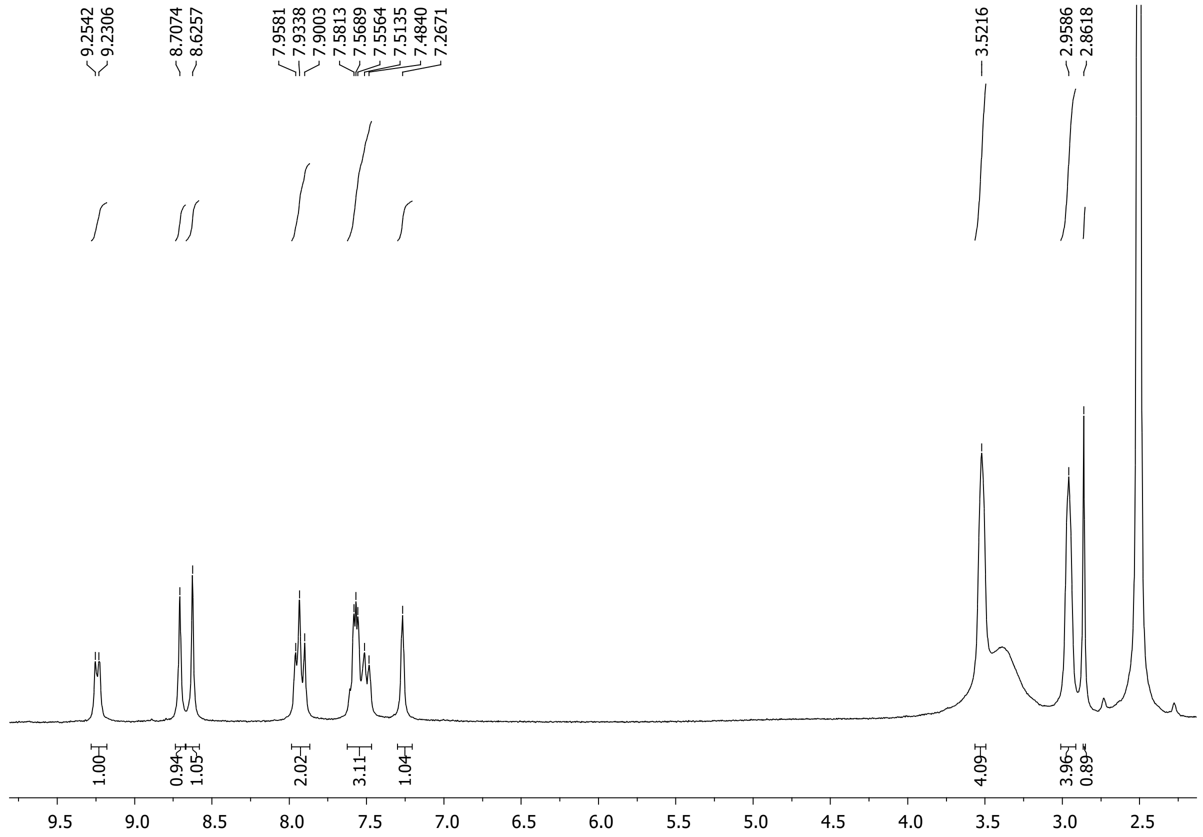

Figure S39. ^{1}H NMR spectrum (DMSO-d_6, 300 MHz) of *11-(piperazin-1-yl)benzo[g]benzo[4,5]imidazo[1,2-a][1,8]naphthyridine-6-carbonitrile **22***

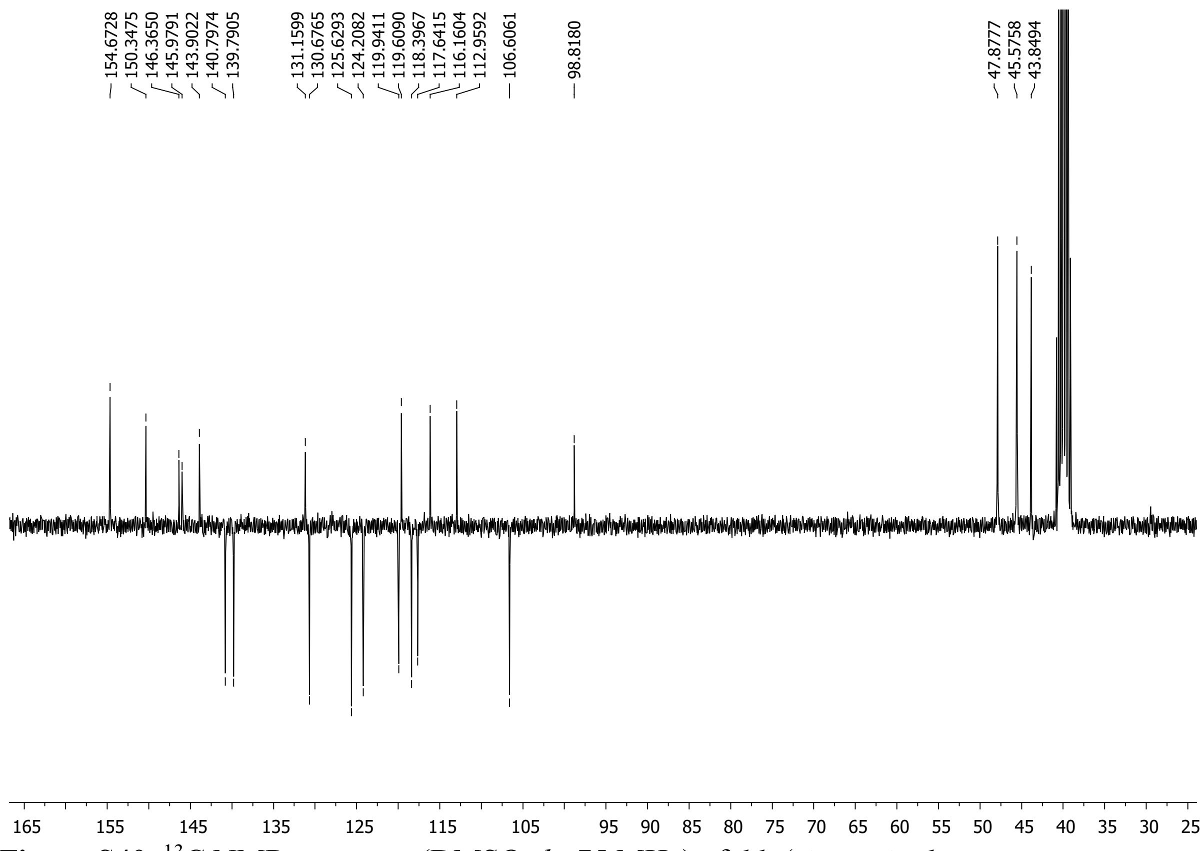

Figure S40. ^{13}C NMR spectrum (DMSO-d_6, 75 MHz) of *11-(piperazin-1-yl)benzo[g]benzo[4,5]imidazo[1,2-a][1,8]naphthyridine-6-carbonitrile* ***22***

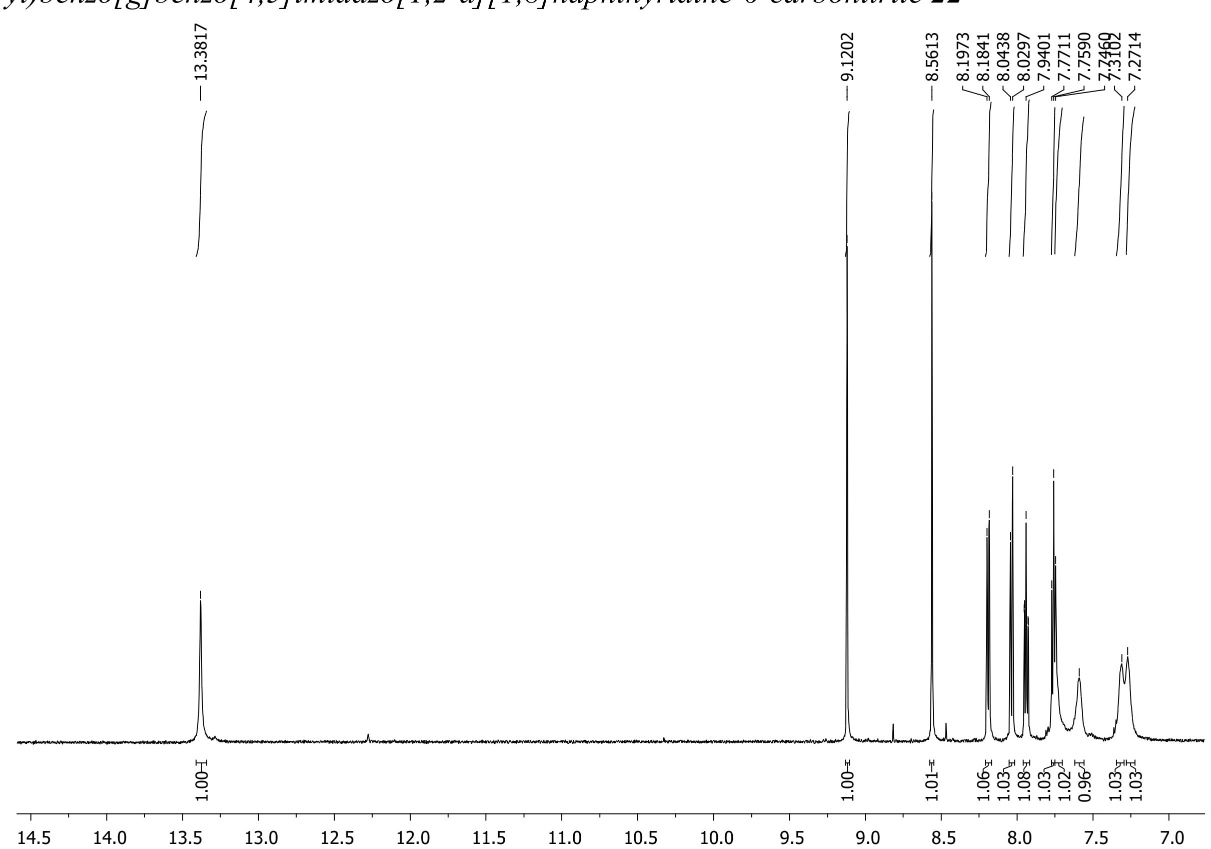

Figure S41. ^{1}H NMR spectrum (DMSO-d_6, 600 MHz) of *(E)-2-(1H-benzo[d]imidazol-2-yl)-3-(2-chloroquinolin-3-yl)acrylonitrile* ***14***

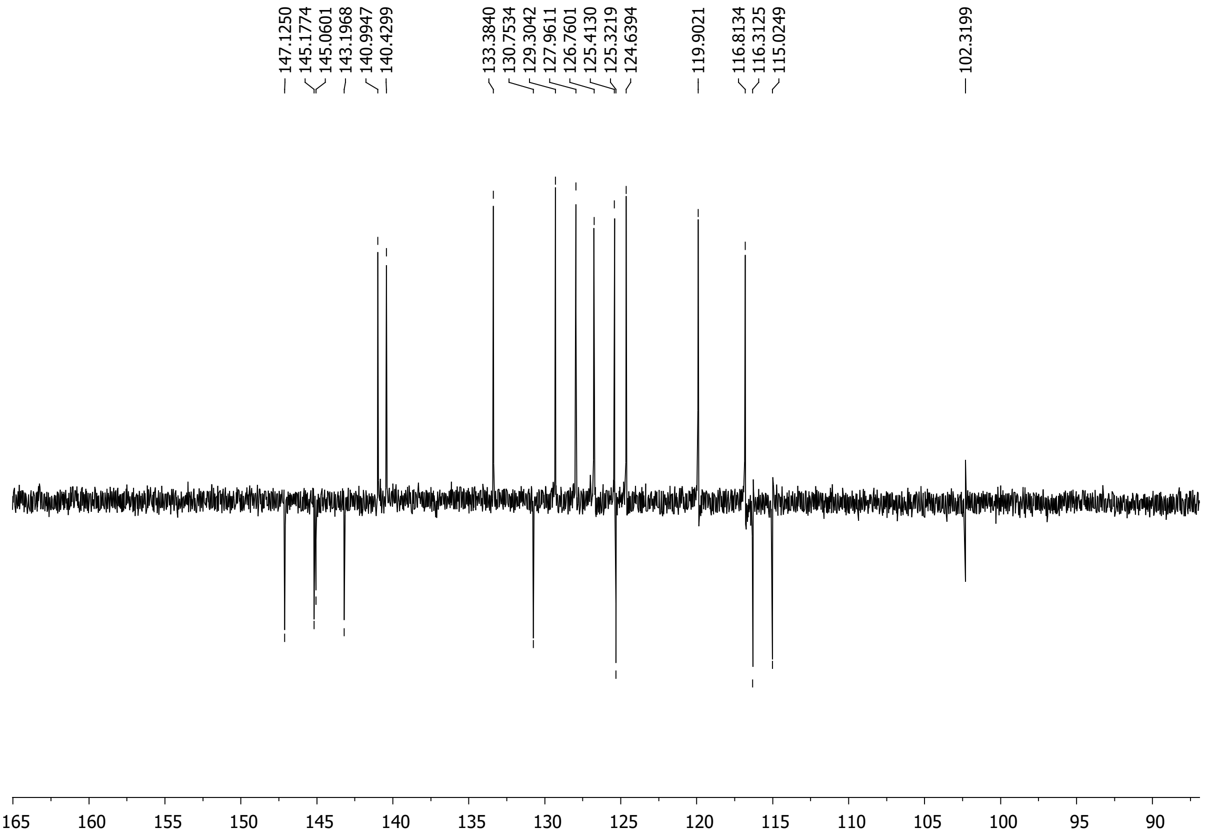

Figure S42. ^{13}C NMR spectrum (DMSO-d_6, 150 MHz) of *(E)-2-(1H-benzo[d]imidazol-2-yl)-3-(2-chloroquinolin-3-yl)acrylonitrile* ***14***

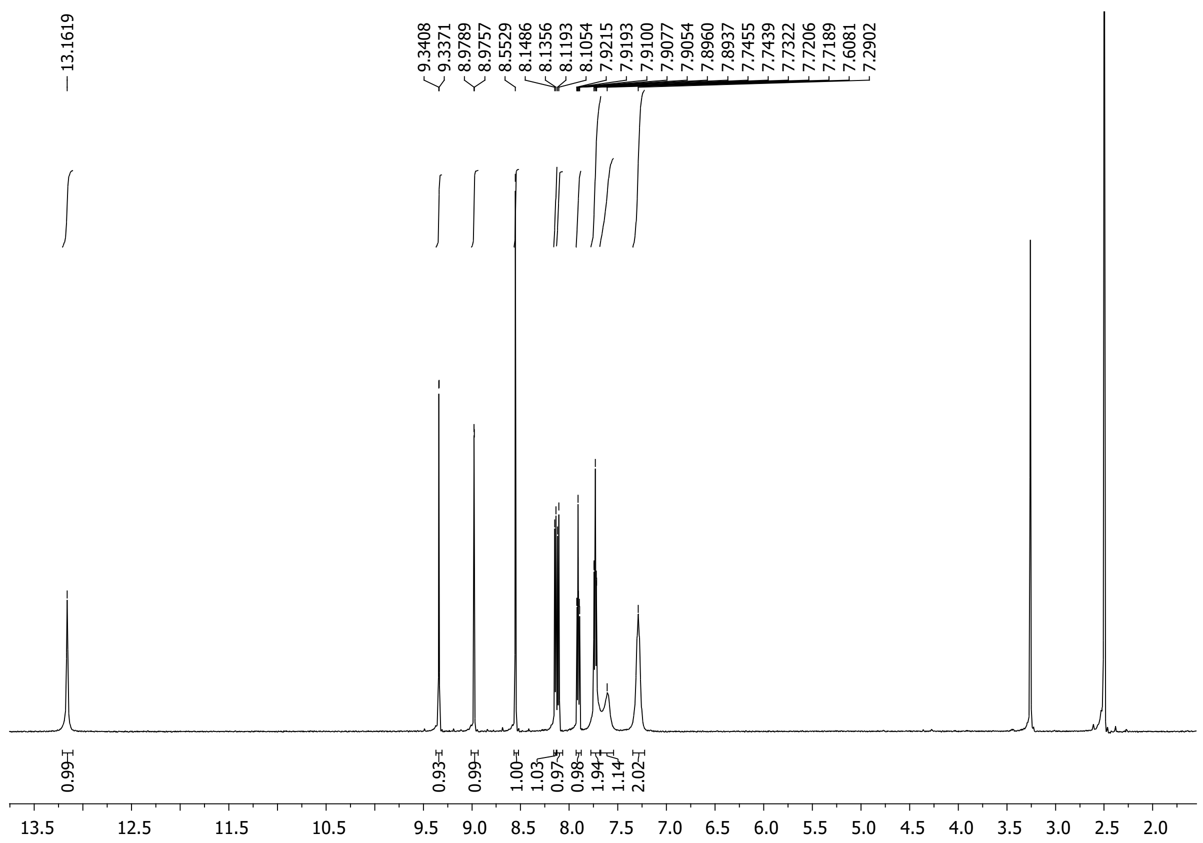

Figure S43. ^{1}H NMR spectrum (DMSO-d_6, 600 MHz) of *(E)-2-(1H-benzo[d]imidazol-2-yl)-3-(quinolin-3-yl)acrylonitrile* ***15***

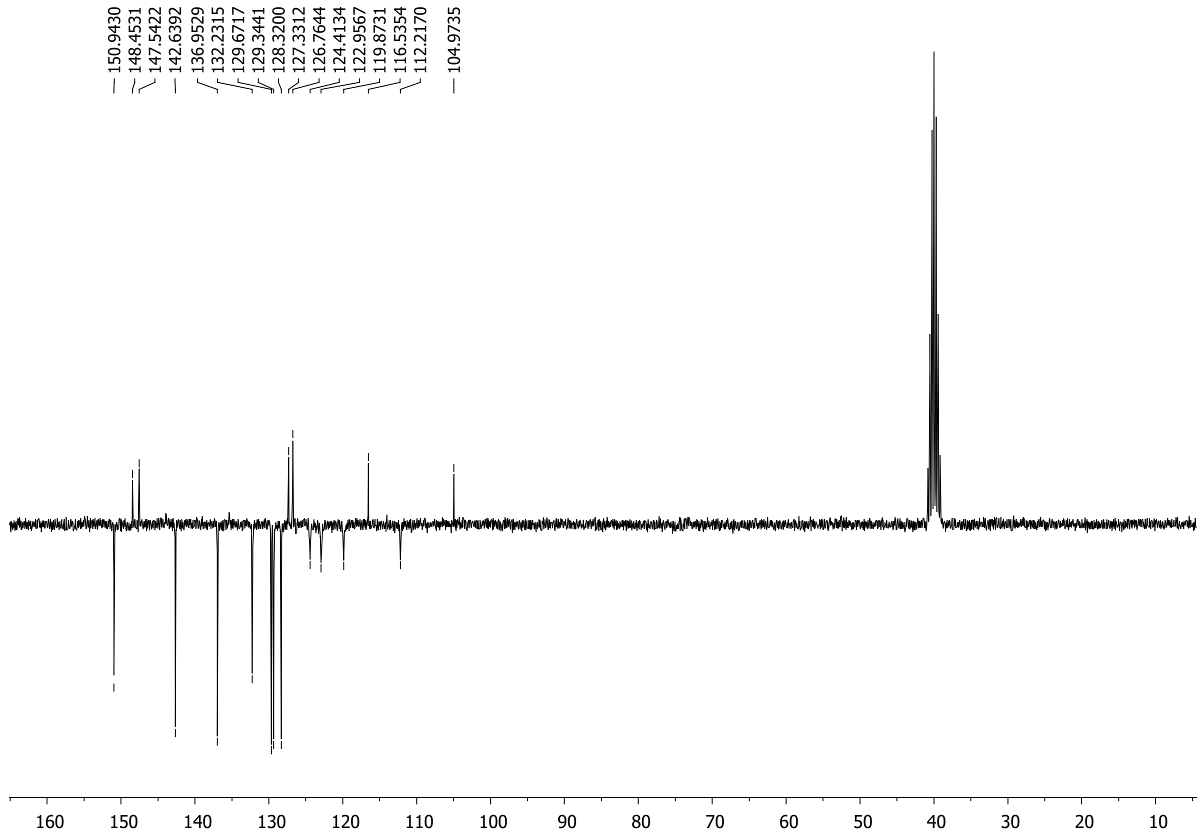

Figure S44. ^{13}C NMR spectrum (DMSO-d_6, 75 MHz) of *(E)-2-(1H-benzo[d]imidazol-2-yl)-3-(quinolin-3-yl)acrylonitrile* ***15***

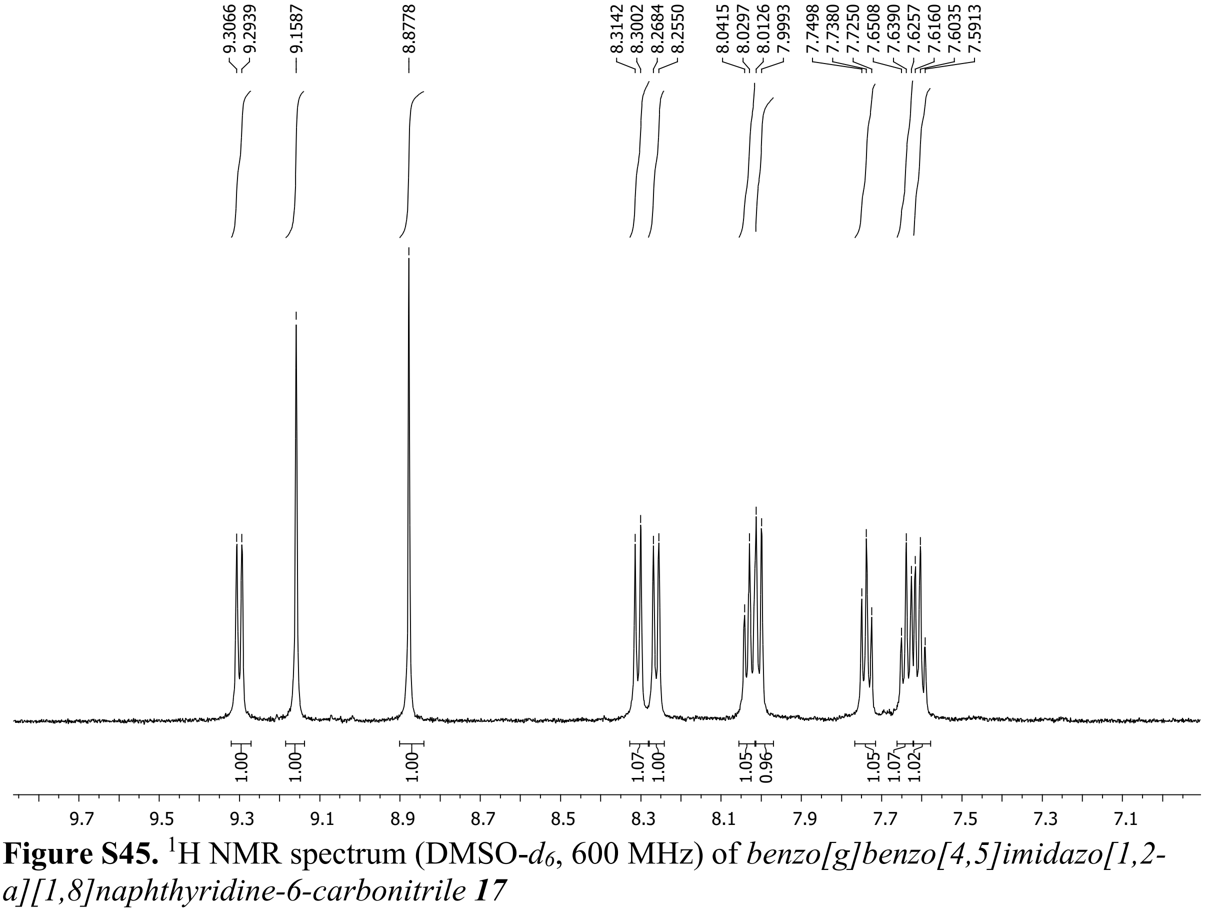

Figure S45. ^{1}H NMR spectrum (DMSO-d_6, 600 MHz) of *benzo[g]benzo[4,5]imidazo[1,2-a][1,8]naphthyridine-6-carbonitrile* ***17***

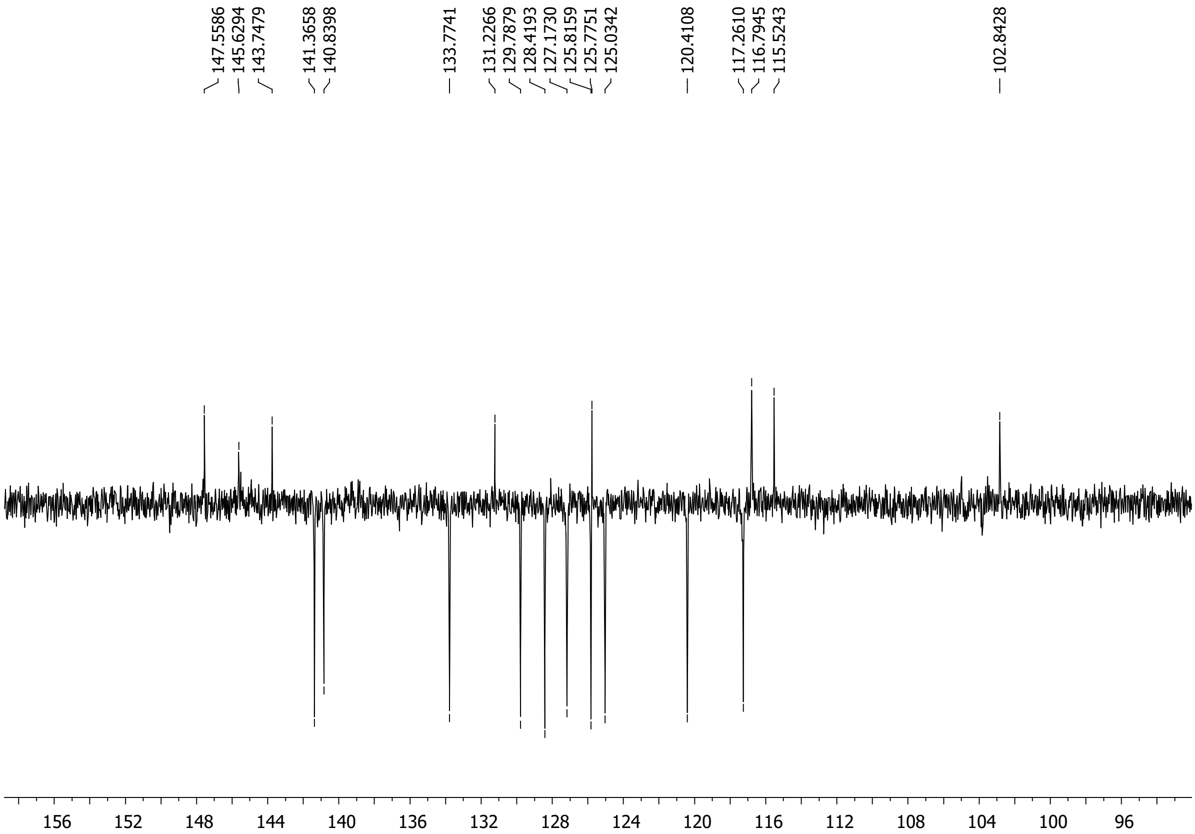

Figure S46. ^{13}C NMR spectrum (DMSO-d_6, 75 MHz) of *benzo[g]benzo[4,5]imidazo[1,2-a][1,8]naphthyridine-6-carbonitrile* ***17***

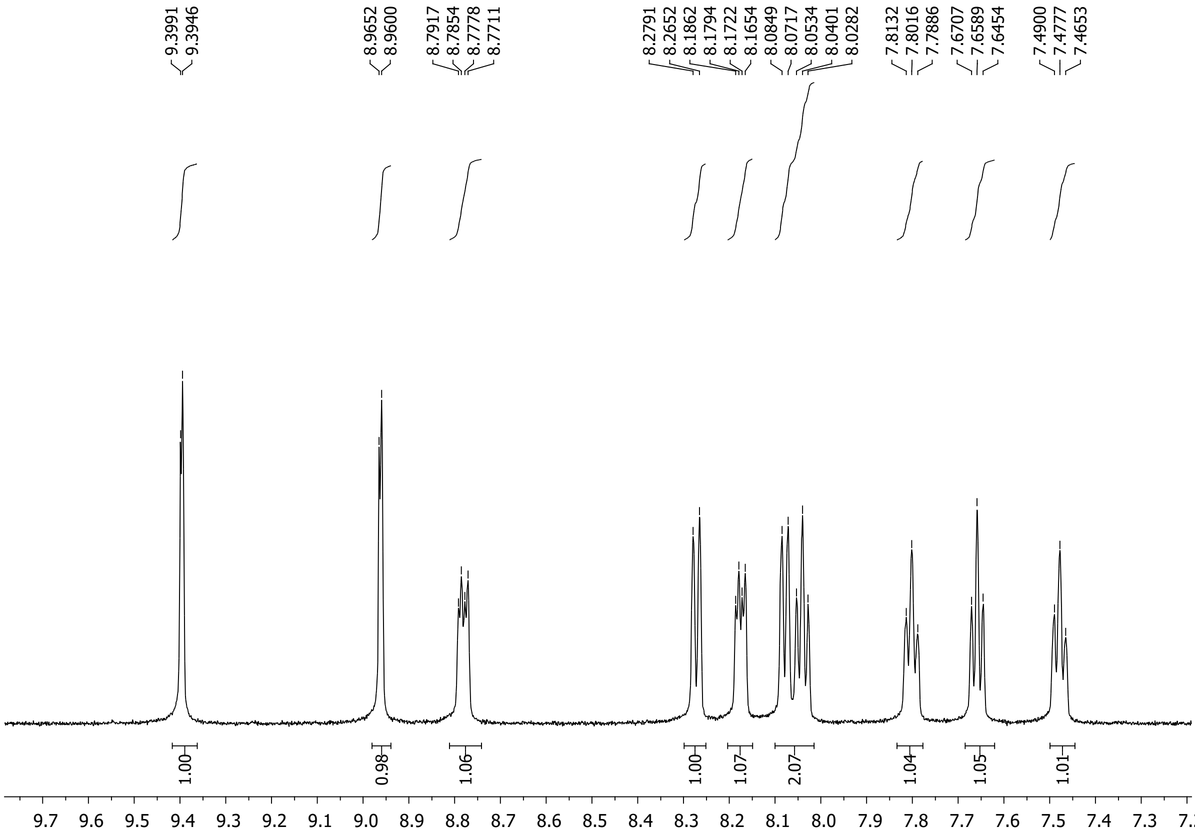

Figure S47. ^{1}H NMR spectrum (DMSO-d_6, 600 MHz) of *benzo[g]benzo[4,5]imidazo[1,2-a][1,5]naphthyridine-7-carbonitrile* ***18***

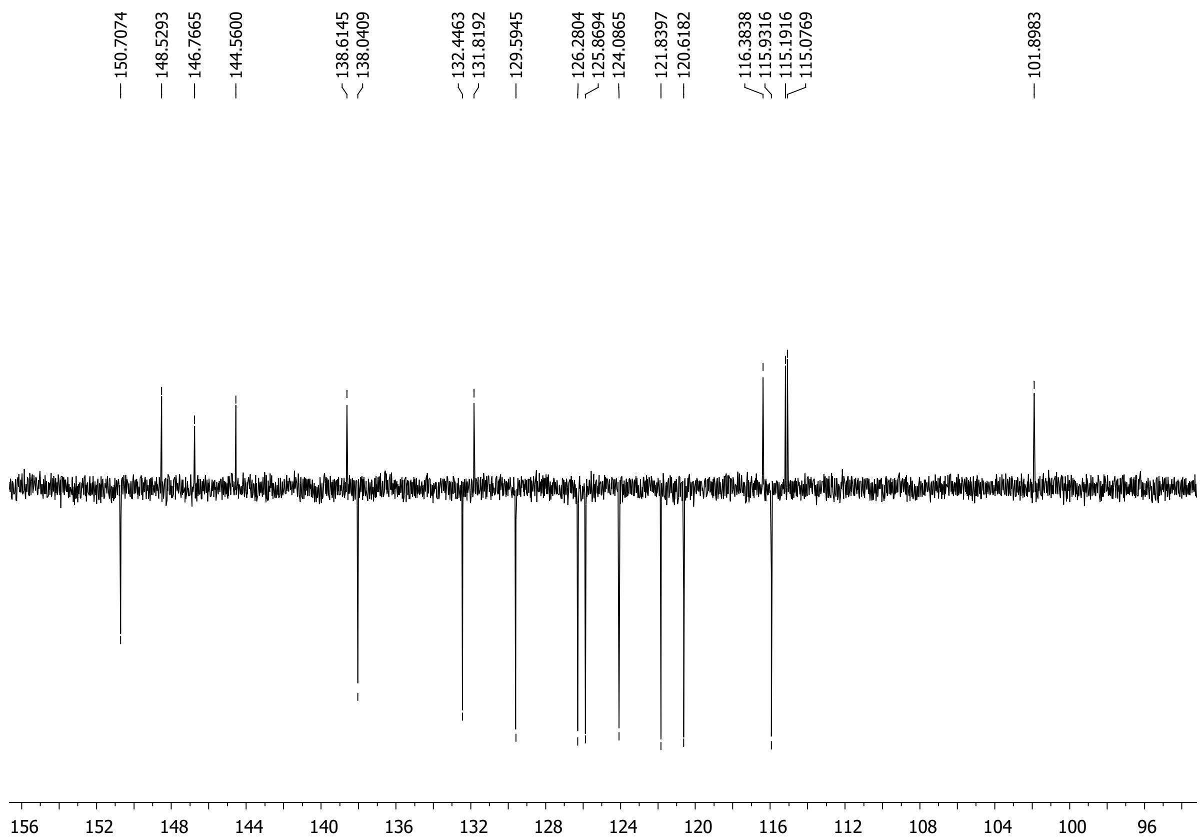

Figure S48. ^{13}C NMR spectrum (DMSO-d_6, 150 MHz) of *benzo[g]benzo[4,5]imidazo[1,2-a][1,5]naphthyridine-7-carbonitrile* ***18***

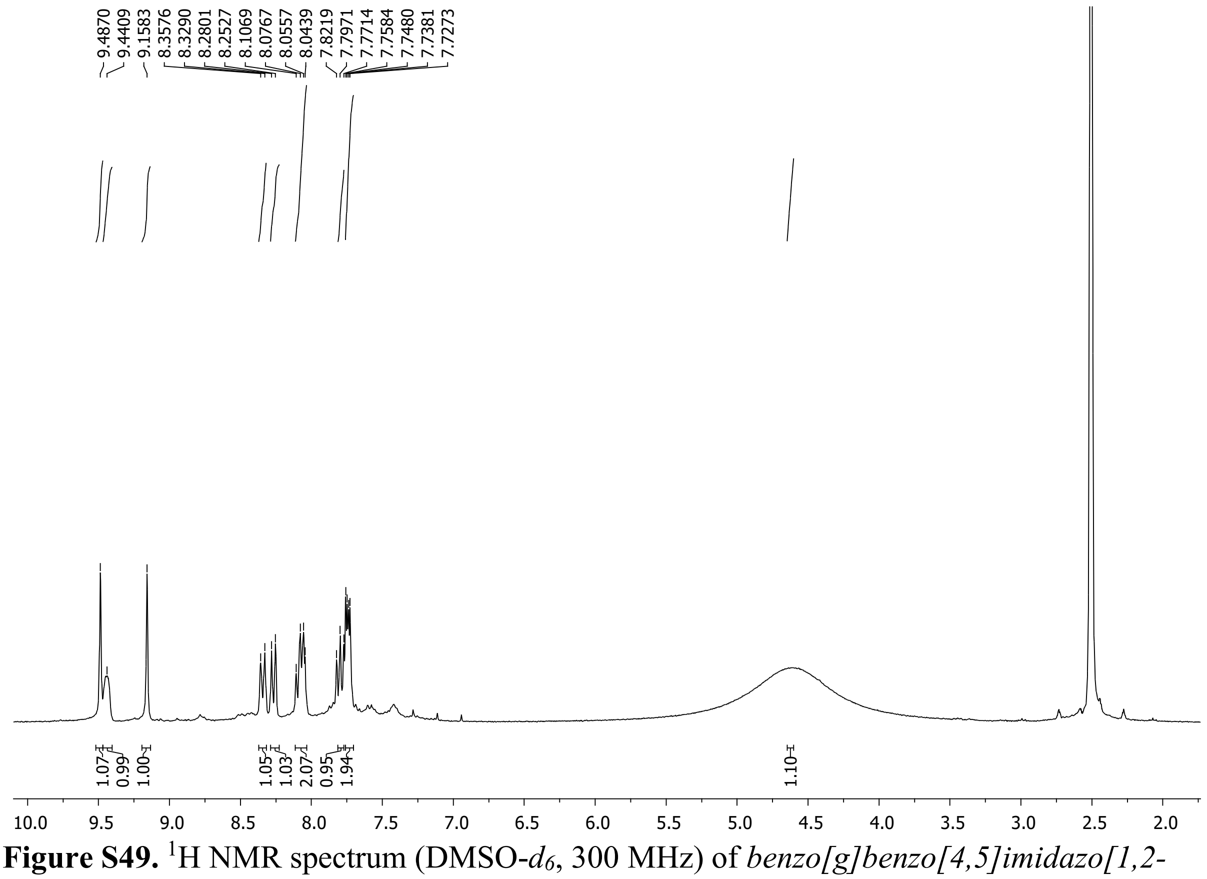

Figure S49. ^{1}H NMR spectrum (DMSO-d_6, 300 MHz) of *benzo[g]benzo[4,5]imidazo[1,2-a][1,8]naphthyridine-6-carboxylic acid* ***23***

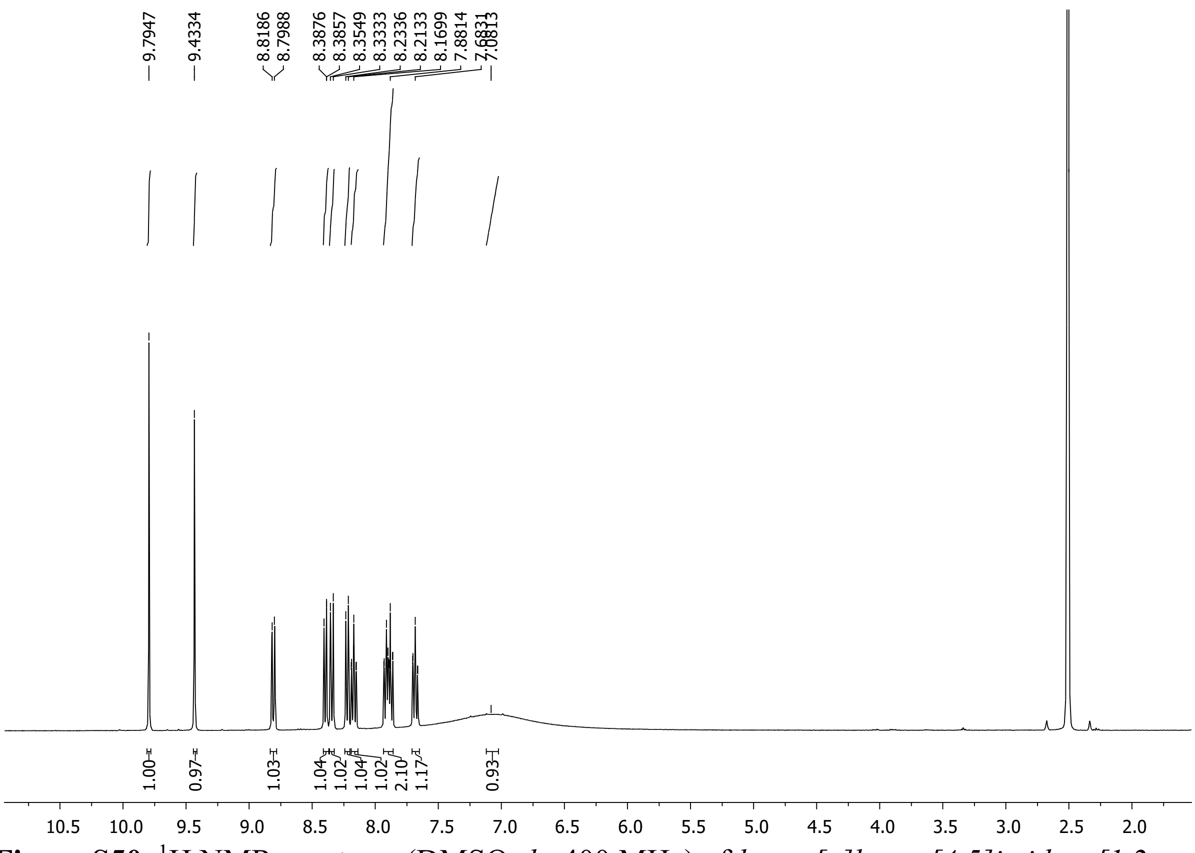

Figure S50. ^{1}H NMR spectrum (DMSO-d_6, 400 MHz) of *benzo[g]benzo[4,5]imidazo[1,2-a][1,5]naphthyridine-7-carboxylic acid* ***27***

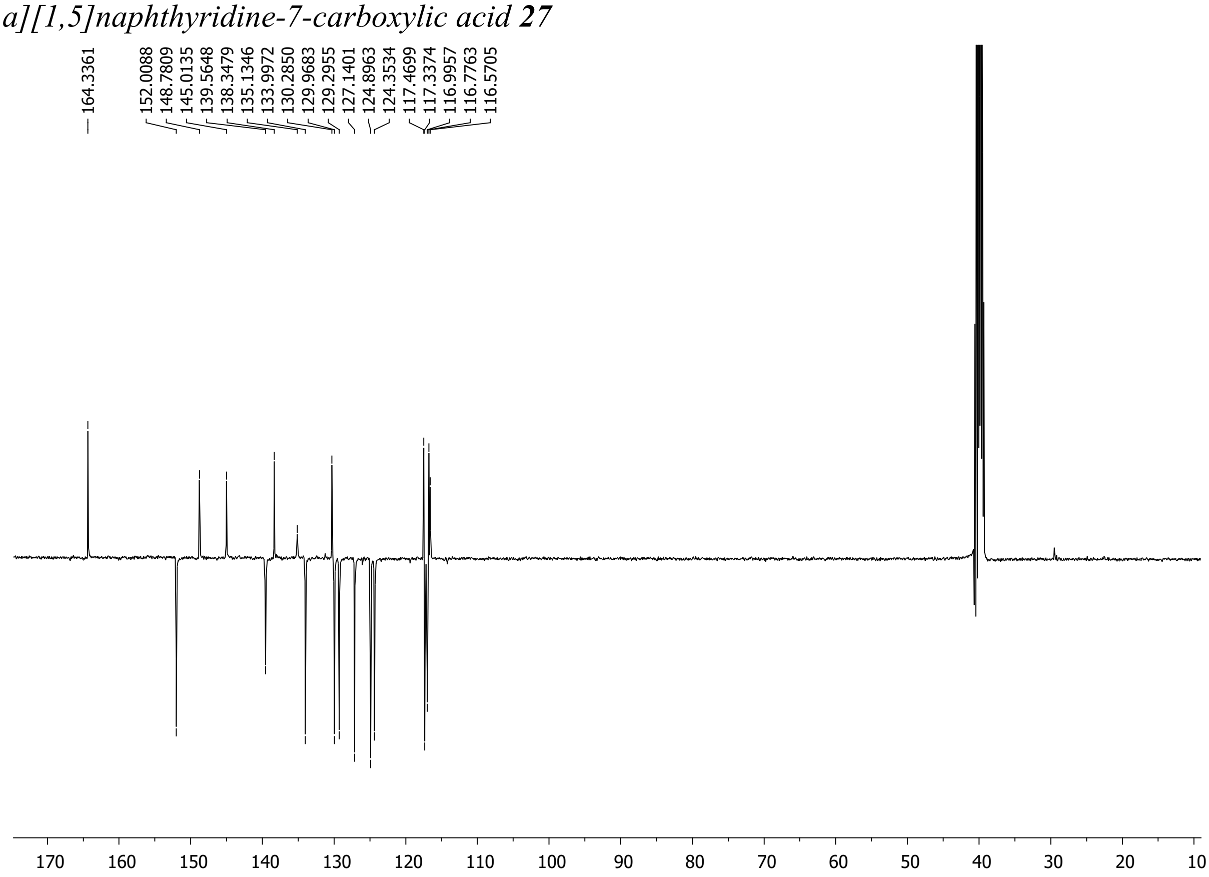

Figure S51. ^{13}C NMR spectrum (DMSO-d_6, 100 MHz) of *benzo[g]benzo[4,5]imidazo[1,2-a][1,5]naphthyridine-7-carboxylic acid* ***27***

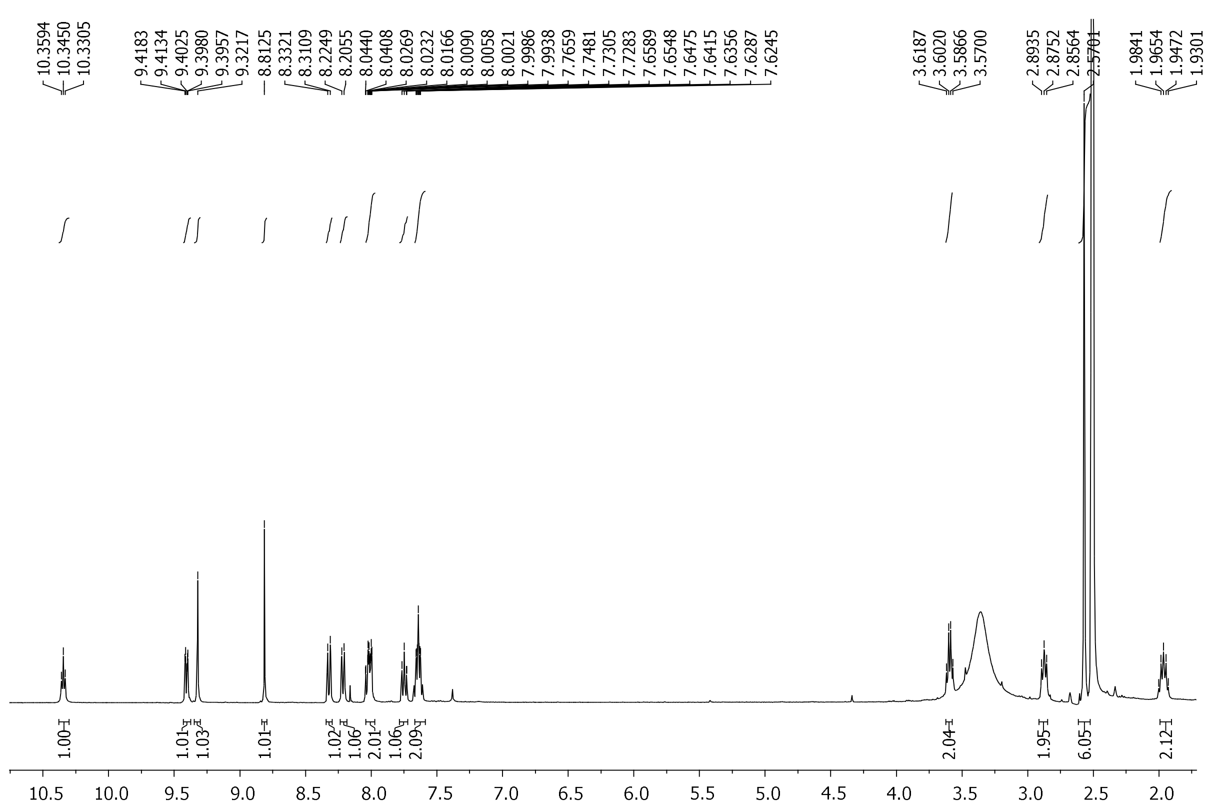

Figure S52. ^{1}H NMR spectrum (DMSO-d_6, 400 MHz) of *N-(3-N,N-(dimethylamino)propyl)benzo[g]benzo[4,5]imidazo[1,2-a][1,8]naphthyridine-6-carboxamide* **25**

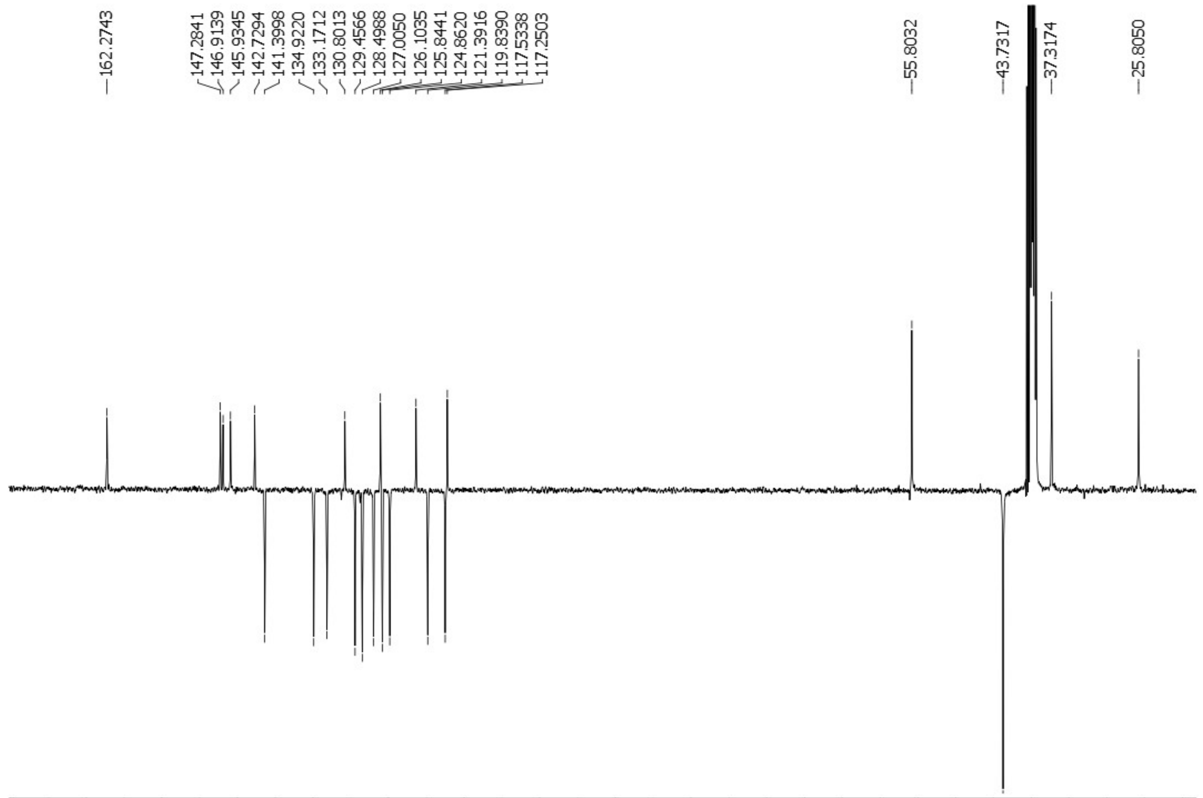

Figure S53. ^{13}C NMR spectrum (DMSO-d_6, 100 MHz) of *N-(3-N,N-(dimethylamino)propyl)benzo[g]benzo[4,5]imidazo[1,2-a][1,8]naphthyridine-6-carboxamide* **25**

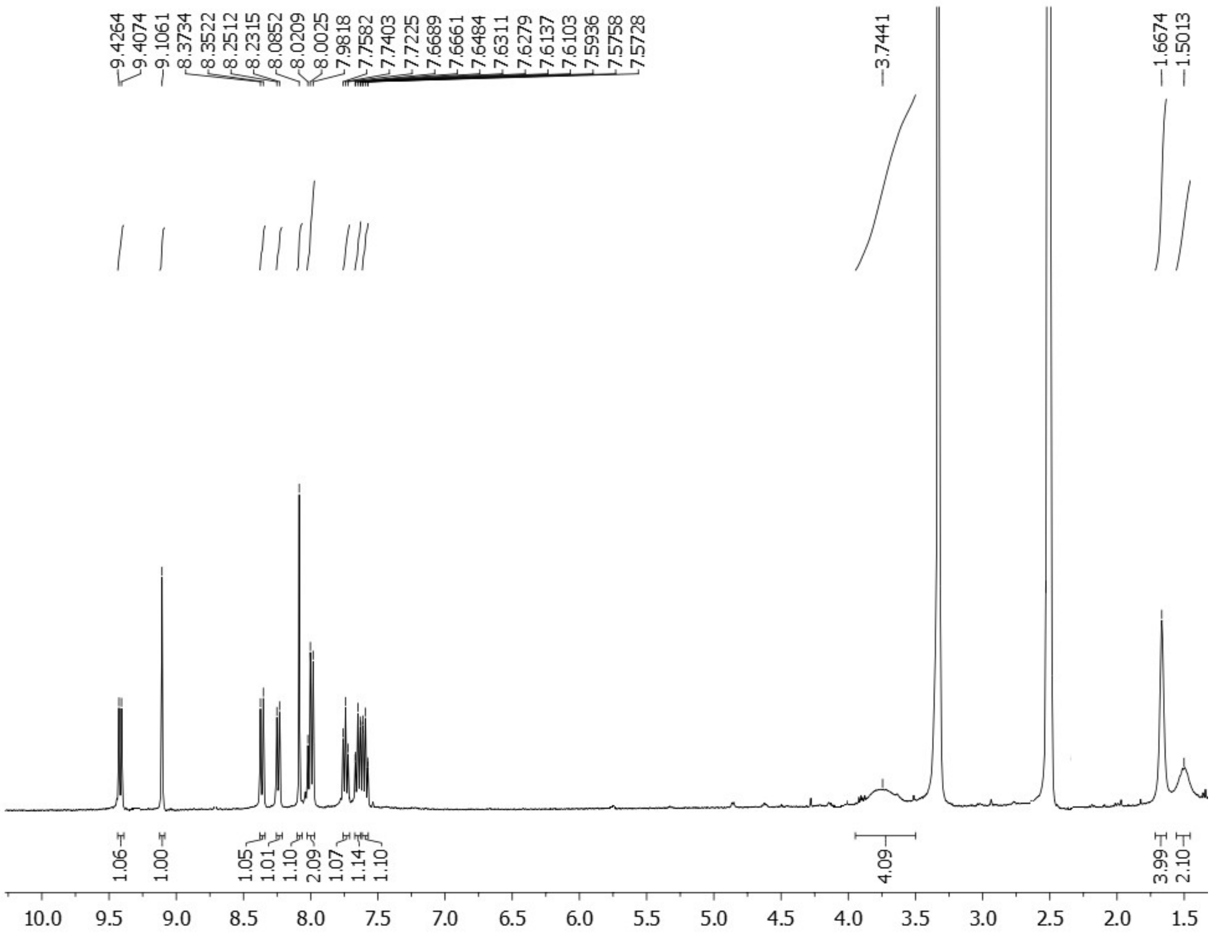

Figure S54. ^{1}H NMR spectrum (DMSO-d_6, 400 MHz) of *benzo[g]benzo[4,5]imidazo[1,2-a][1,8]naphthyridin-6-yl(piperidin-1-yl)methanone* **26**

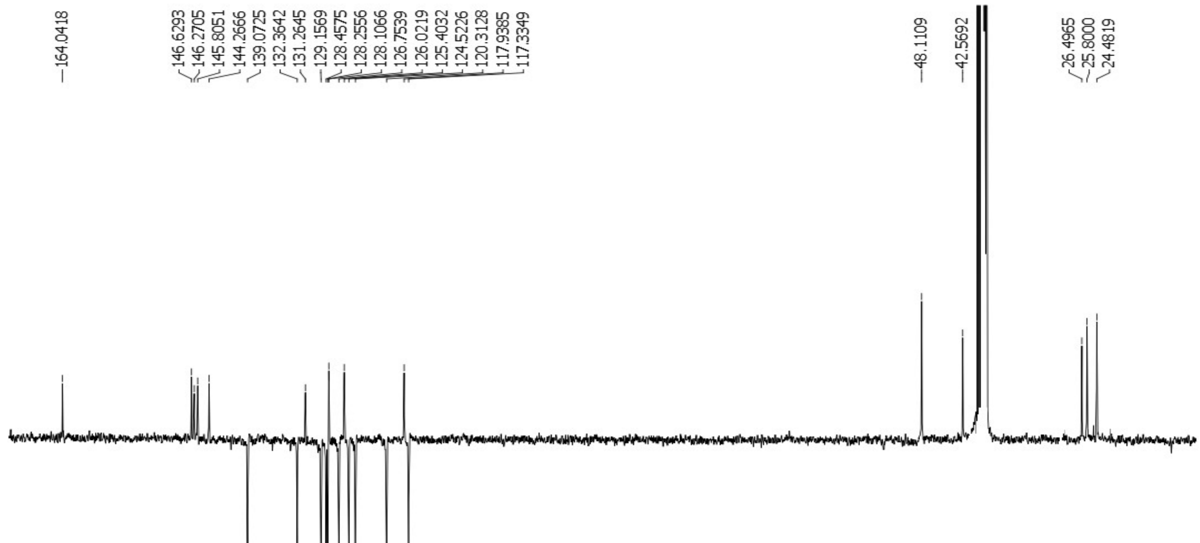

Figure S55. ^{13}C NMR spectrum (DMSO-d_6, 100 MHz) of *benzo[g]benzo[4,5]imidazo[1,2-a][1,8]naphthyridin-6-yl(piperidin-1-yl)methanone* **26**

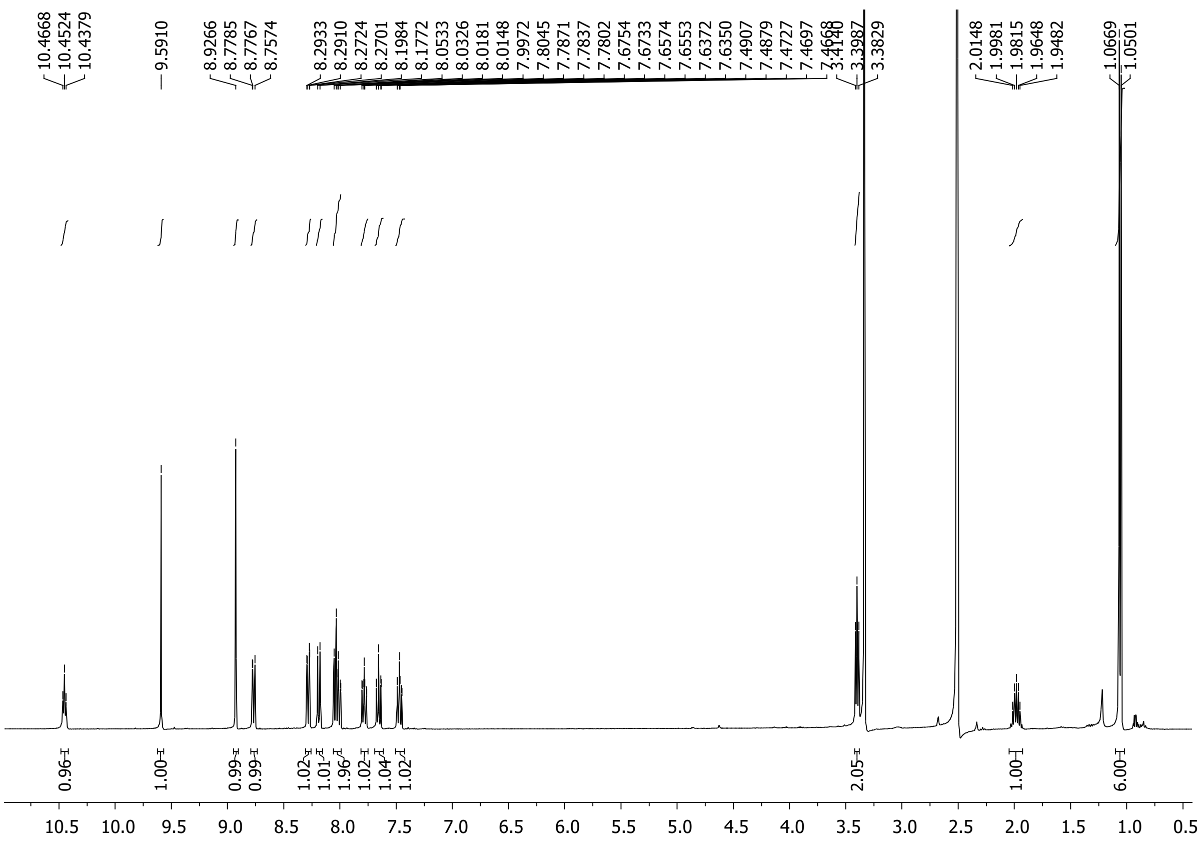

Figure S56. ^{1}H NMR spectrum (DMSO-d_6, 400 MHz) of *N-isobutylbenzo[g]benzo[4,5]imidazo[1,2-a][1,5]naphthyridine-7-carboxamide* **29**

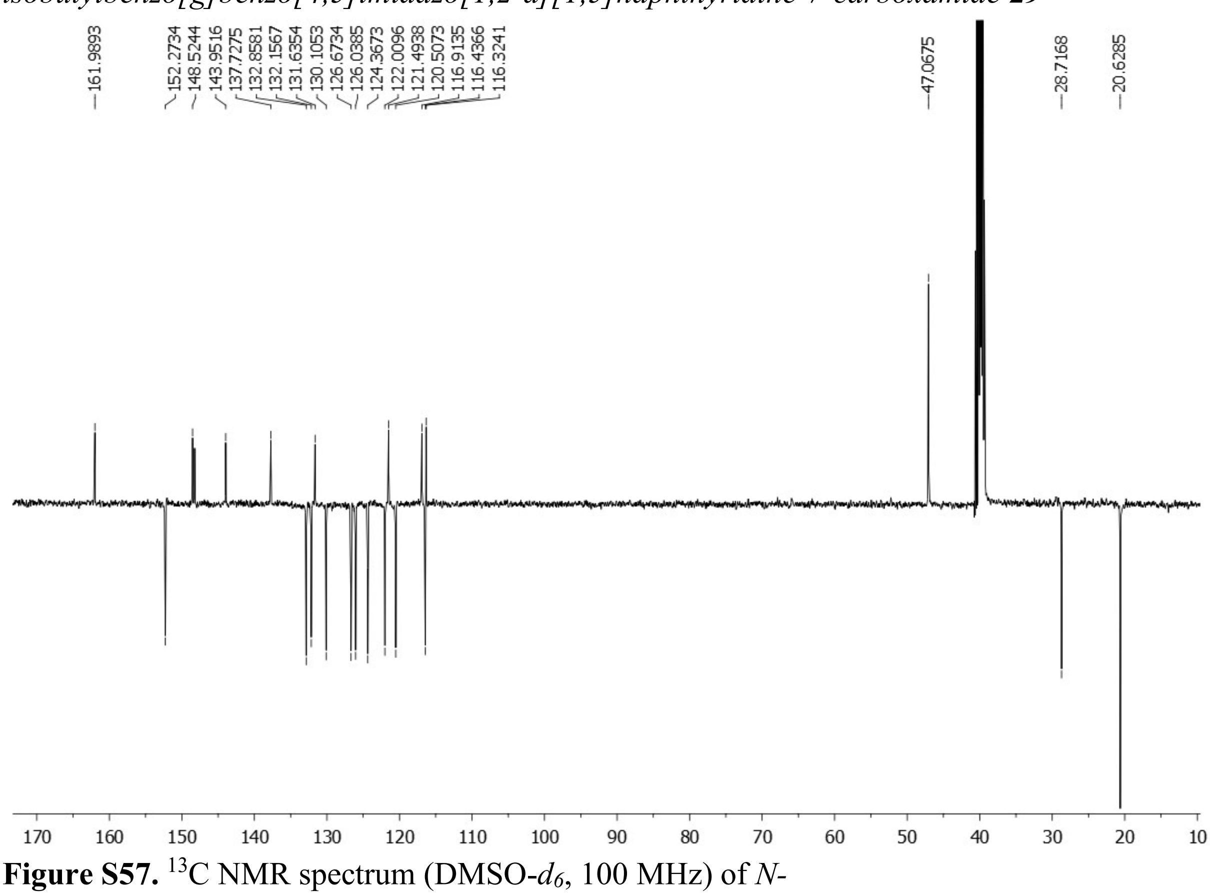

Figure S57. ^{13}C NMR spectrum (DMSO-d_6, 100 MHz) of *N-isobutylbenzo[g]benzo[4,5]imidazo[1,2-a][1,5]naphthyridine-7-carboxamide* **29**

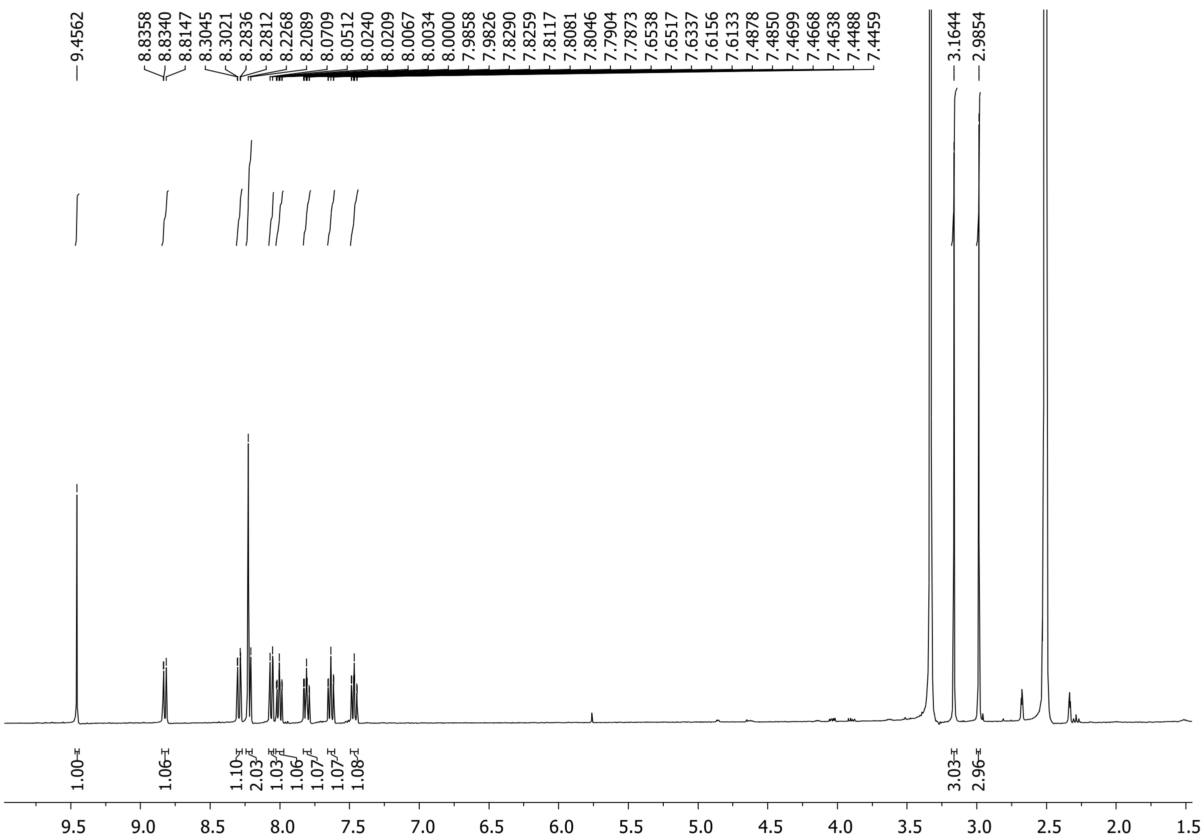

Figure S58. ^{1}H NMR spectrum (DMSO-d_6, 400 MHz) of *N,N-dimethylbenzo[g]benzo[4,5]imidazo[1,2-a][1,5]naphthyridine-7-carboxamide* **30**

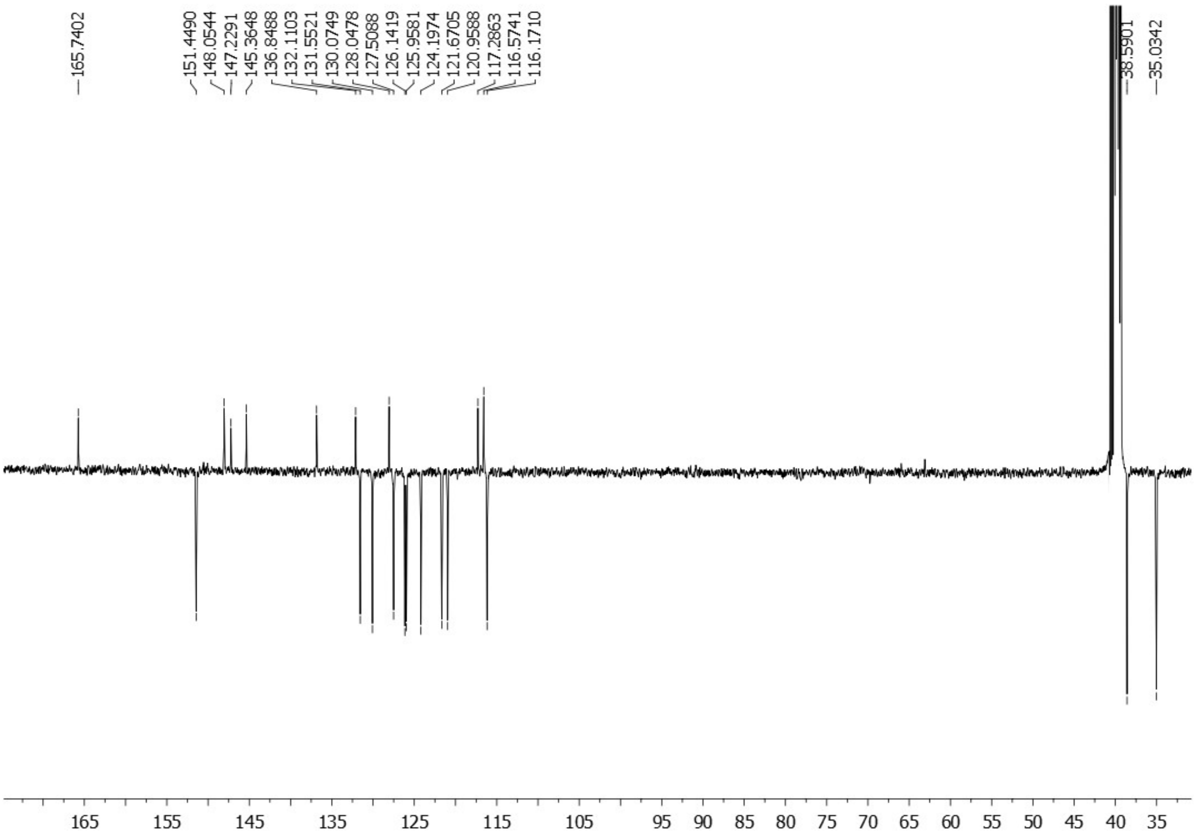

Figure S59. ^{13}C NMR spectrum (DMSO-d_6, 100 MHz) of *N,N-dimethylbenzo[g]benzo[4,5]imidazo[1,2-a][1,5]naphthyridine-7-carboxamide* **30**
